# PDIA3 Expression in Glioblastoma Modulates Macrophage/Microglia Pro-Tumor Activation

**DOI:** 10.3390/ijms21218214

**Published:** 2020-11-03

**Authors:** Marta Chiavari, Gabriella Maria Pia Ciotti, Francesco Canonico, Fabio Altieri, Pedro Miguel Lacal, Grazia Graziani, Pierluigi Navarra, Lucia Lisi

**Affiliations:** 1 Dipartimento di Bioetica e Sicurezza, Sezione di Farmacologia—Catholic University Medical School, 00168 Rome, Italy; m.chiavari@hotmail.it (M.C.); gabriella.ciotti@hotmail.it (G.M.P.C.); pierluigi.navarra@unicatt.it (P.N.); lucia.lisi@unicatt.it (L.L.); 2 Dipartimento di Scienze Cardiovascolari e Toraciche, Fondazione Policlinico Universitario A. Gemelli IRCCS, Catholic University Medical School, 00168 Rome, Italy; francesco.canonico@unicatt.it; 3 Dipartimento di Scienze Biochimiche “A. Rossi Fanelli”, Sapienza University, P.le A. Moro 5, 00185 Rome, Italy; fabio.altieri@uniroma1.it; 4 IDI-IRCCS, Via dei Monti di Creta 104, 00167 Rome, Italy; p.lacal@idi.it; 5 Department of Systems Medicine, University of Rome Tor Vergata, Via Montpellier 1, 00133 Rome, Italy; 6 Fondazione Policlinico Universitario A. Gemelli IRCCS, 00168 Rome, Italy

**Keywords:** PDIA3, STAT3, IL6, microglia, glioma, punicalagin

## Abstract

The glioblastoma (GB) microenvironment includes cells of the innate immune system identified as glioma-associated microglia/macrophages (GAMs) that are still poorly characterized. A potential role on the mechanisms regulating GAM activity might be played by the endoplasmic reticulum protein ERp57/PDIA3 (protein disulfide-isomerase A3), the modulation of which has been reported in a variety of cancers. Moreover, by using The Cancer Genome Atlas database, we found that overexpression of PDIA3 correlated with about 55% reduction of overall survival of glioma patients. Therefore, we analyzed the expression of ERp57/PDIA3 using specimens obtained after surgery from 18 GB patients. Immunohistochemical analysis of tumor samples revealed ERp57/PDIA3 expression in GB cells as well as in GAMs. The ERp57/PDIA3 levels were higher in GAMs than in the microglia present in the surrounding parenchyma. Therefore, we studied the role of PDIA3 modulation in microglia–glioma interaction, based on the ability of conditioned media collected from human GB cells to induce the activation of microglial cells. The results indicated that reduced PDIA3 expression/activity in GB cells significantly limited the microglia pro-tumor polarization towards the M2 phenotype and the production of pro-inflammatory factors. Our data support a role of PDIA3 expression in GB-mediated protumor activation of microglia.

## 1. Introduction

Glioblastoma (GB—grade IV glioma) is the most aggressive and common cancer of the central nervous system in adults, with over 10,000 new cases in the United States per year [1]. The first-line treatment consists in a multimodal approach with surgical resection, when possible, followed by radiation therapy, plus concomitant and adjuvant chemotherapy with temozolomide [2]. However due to its invasiveness and infiltrative behavior, GB is characterized by a median overall survival of only 14–16 months. Therefore, this tumor still represents an unmet medical need and it is necessary to identify novel targets, both in the tumor and its microenvironment, to develop more effective and targeted therapies.

The GB tumor microenvironment includes cells of the innate immune system identified as glioma-associated microglia/macrophages (GAMs) that represent the largest population infiltrating the tumor [3]. GAMs possess different dynamic states of activation: the M1 state, which is tumor suppressive, and the M2 state, which is tumor supportive, contributing to tumor growth. These two different phenotypes are thought to reflect a spectrum of plastic functional conditions rather than a set of discrete activation states [4,5]. In addition, microglia may possess multiple context-dependent properties at the same time. However, although it is known that, between GAMs and GB cells, there is a reciprocal interaction, the role of this cross-talk on GB progression is still poorly characterized.

**Figure 1:**
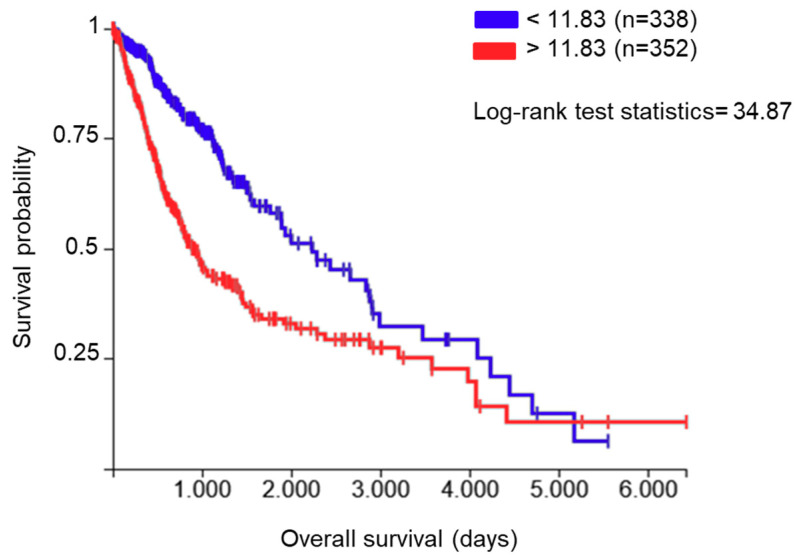
Correlation between protein disulfide-isomerase A3 (PDIA3) expression in glioma tissues and patients’ survival: data from The Cancer Genome Atlas (TCGA) lower grade glioma and glioblastoma (GB) were examined for the correlation between overall survival and PDIA3 gene expression levels. A total of 690 patients (male and female combined) were divided into two groups with low (blue lines, *n* = 338) and high (red lines, *n* = 352) PDIA3 expression. Kaplan–Meier curves were generated to test for correlation by Log-rank (Mantel–Cox) test. *p* < 0.00001.

The endoplasmic reticulum (ER) protein ERp57, also known as protein disulfide-isomerase A3 (PDIA3), GRP58, or ERp60, is a thiol oxidoreductase and is a member of the protein disulfide isomerase (PDI) family that counts several members with different localization and functionality. These proteins have a thioredoxin (TRX)-like domain and are mostly localized in the ER [6,7]; however, different localizations have been reported [8]. The first and main function of PDIA3 is related to glycoprotein folding in the ER [9]. In fact, in association with the lectins calnexin and calreticulin, PDIA3 regulates the folding of newly synthesized cysteine-rich glycoproteins through the formation and disruption of disulfide bonds between cysteine residues [10,11]. Beyond its activity in the ER, PDIA3 plays additional functions which depend on its cellular localization. In the cell membrane, for example, PDIA3 acts as a membrane receptor for 1α, 25-dihydroxy-vitamin D3 [12,13]. In the cytoplasm, it co-localizes with NF-κB or mammalian target of rapamycin (mTOR), forming, in the latter case, a complex that has been implicated in various developmental processes [14]. In the nucleus, PDIA3 directly interacts with DNA or enhances the binding to DNA of the signal transducer and activator of transcription 3 (STAT3) complex and facilitates the nuclear import and export of this transcription factor [15,16,17]. Therefore, the various subcellular localizations and binding partners of PDIA3 affect numerous physiological processes and diseases. In fact, different alterations in the regulatory pathways that modulate PDIA3 activity/expression have been reported in multiple pathologies, including neurodegenerative diseases [18,19] and cancer [20,21,22]. By analyzing the role of PDIA3 in GB survival using The Cancer Genome Atlas (TCGA) database (https://xenabrowser.net/, accessed on 2 November 2020), 352 out of 690 patients (dataset: TCGA lower grade glioma and GB) showed increased PDIA3 expression in the tumor tissue that was associated with an approximately 55% reduction of overall survival compared to patients with lower PDIA3 levels (Figure 1).

Moreover, PDIA3 expression level has been evaluated as a biomarker in several conditions [23,24] and proposed as a potential novel pharmacological target.

PDIA3 signaling has a natural pharmacological inhibitor, punicalagin (PUN), a tannin deriving from the secondary metabolism of different plants, which represents the known largest molecular weight polyphenol found in pomegranates (Punica granatum), Terminalia catappa, Terminalia myriocarpa, and Combretum molle. PUN possesses several properties among which anti-inflammatory activity; moreover, recent studies have demonstrated its ability to bind PDIA3 and inhibit its redox activity [25].

In the last years, our group has investigated the role of GAMs in murine and human models of GB microenvironment, looking at different regulatory molecules involved in GAM polarization and activation status [26,27,28,29,30]. In the present study, we have evaluated both in GB cells and microglia the expression of PDIA3 in GB specimens collected from patients after surgical resection of the tumor. In addition, we studied in vitro microglia–glioma interaction to determine the role of PDIA3 in favoring the cross-talk between GB cells and microglia. By means of gene silencing or pharmacological inhibition using PUN, we demonstrated that PDIA3 is involved in the pro-tumor activity of GB cells and GAMs.

## 2. Results

### 2.1. PDIA3 Expression in GB Specimens

**Figure 2:**
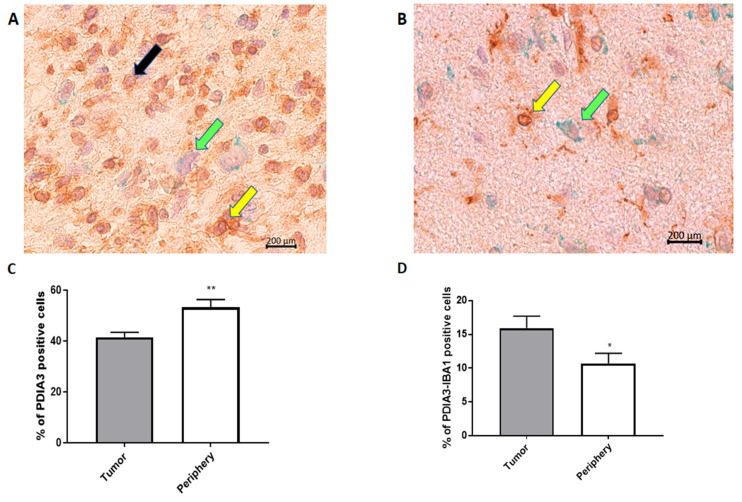
PDIA3 staining in human GB specimens: the photographs show a representative tumor field (**A**) and a representative positive parenchyma field (**B**) stained for PDIA3 and IBA1 (magnification: 40×). Yellow arrows point to double IBA1/PDIA3 stained cells, whereas black and green arrows point to IBA1 and PDIA3 positive cells, respectively. (**C**,**D**) The histograms represent the percentage of PDIA3 positive (**C**) or PDIA3/IBA1 double-positive cells (**D**) in human GB specimens, taking into account the tumor or the parenchyma regions (periphery). Data are expressed as mean ± SEM of samples collected from 18 patients. Tumor versus periphery, * *p* < 0.05; ** *p* < 0.01.

In order to analyze the PDIA3 expression pattern in GB, we examined the tumor and surrounding parenchyma in tissue specimens collected after tumor surgical removal from 18 patients diagnosed with GB. Unexpectedly, the tumor tissue presented a significantly lower percentage of PDIA3 stained cells in comparison with the surrounding parenchyma (Figure 2A–C).

In fact, in the parenchyma, >50% of the cells were positive for PDIA3, while in the tumor, the percentage of cells expressing PDIA3 was about 40% (Figure 2C). Interestingly, the staining of PDIA3 was not the same for all cell types: some cells presented nuclear membrane staining, whereas other cells stained positively also in the cytoplasm (Figure 2A,B). These data suggest that PDIA3 is expressed by GB cells as well as by cells of the tumor-associated microenvironment. Therefore, since microglia represent the main cell type that infiltrates the tumor we analyzed the expression of PDIA3 in GAMs. By double staining for PDIA3 and IBA1 (a macrophage-microglia marker), we found that the percentage of microglia macrophages expressing PDIA3 present in the tumor was significantly higher than that in the parenchyma (Figure 2D). In fact, taking into account only the microglia-macrophage cell population, in the tumor, 15% of the IBA1 positive cells also expressed PDIA3, whereas in the parenchyma, the percentage of double positive cells was 10%. In the parenchyma and tumor of all the 18 patients analyzed, the percentage of cells positive for IBA1 was similar [30]. Figure S1 shows representative images of the tumor and parenchyma for all 18 patients (Figure S1).

### 2.2. Influence of PDIA3 Expression on the Inflammatory Response and Viability of Human GB Cells

**Figure 3:**
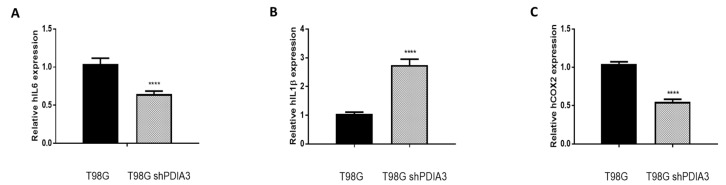
Effects of PDIA3 silencing on IL6, IL1β, and COX2 in human T98G cells: transcript detection was performed by qRT-PCR. Results indicate relative mRNA expression and are the mean ± SEM of three independent determinations. Data show a comparison between (**A**) IL6, (**B**) IL1β, and (**C**) COX2 gene expression of control T98G (calibrator) versus PDIA3-silenced (shPDIA3) T98G cells. **** *p* < 0.0001.

In order to study the effects of PDIA3 on the release of chemokines and cytokines and on the viability of GB cells, the PDIA3 gene was silenced in glioma cells or PDIA3 activity was inhibited by the pharmacological inhibitor PUN. Initially, we measured PDIA3 gene expression in two human GB cell lines, T98G and U87MG, by qRT-PCR. Results indicated that T98G cells express a two-fold higher PDIA3 gene expression; therefore, T98G was chosen to perform subsequent experiments (Figure S2A). In these cells, previous studies have shown that, in T98G, IL6, IL1β, and COX2 are activated after a pro-inflammatory stimulus [28]. Therefore, the effect of the inhibition of PDIA3 expression by siRNA (resulting in about 35% decrease of PDIA3 mRNA and protein, Figure S2B,C) on the expression of these genes was investigated. A 40% reduction in IL6 and COX2 gene expression and an increment of more than 2-fold in IL1β expression was observed in PDIA3 gene silenced cells, compared to control T98G cells (Figure 3).

**Figure 4:**
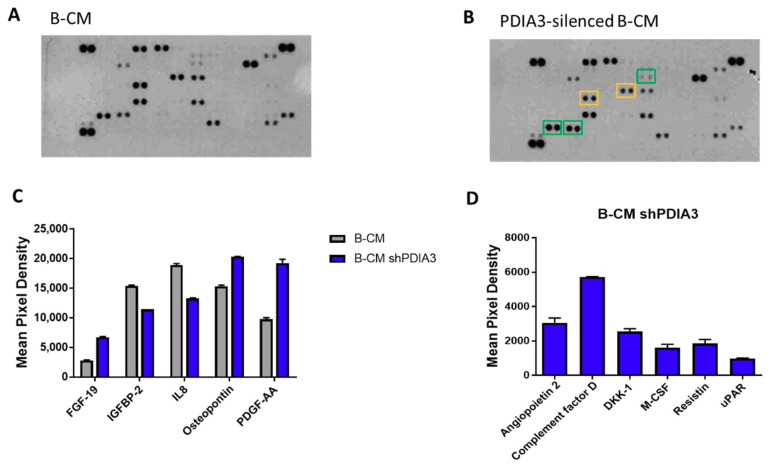
Semi-quantitative analysis of multiple cytokines, chemokines, growth factors, and other soluble proteins in cell culture media of glioma cells: (**A**,**B**) Results of protein analysis in B-Conditioned Medium (CM) collected from control (**A**) and PDIA3-silenced (**B**) T98G cells. Yellow squares and green squares indicate the downregulated factors and upregulated factors, respectively. (**C**) Semi-quantitative analysis of the released proteins showing a statistically significant increase or decrease with *p* < 0.05. (**D**) Proteins whose expression was induced after PDIA3 silencing. Data are the mean ± SD (4). For a complete list of proteins that are modified, see Supplementary Figure S3A.

In addition, using a proteome profiler human cytokine kit, we evaluated the release of a panel of 105 cytokines, chemokines, growth factors, and other soluble proteins in the culture supernatant from both PDIA3-silenced and control T98G cells under basal conditions (hereafter referred to as B-CM (conditioned medium) that mimics an initial stage of GB pathology) (Figure 4A,B). Downregulation of PDIA3 expression resulted in a significant increase of fibroblast growth factor (FGF-19), platelet-derived growth factor (PDFG-AA), and osteopontin and a significant decrease of insulin-like growth factor-binding protein 2 (IGFBP2) and IL8 (Figure 4C). In addition, silencing of PDIA3 in T98G cells induced the release of proteins that were not secreted by control T98G, such as angiopoietin 2, complement factor D, DKK-1, MCSF, resistin, and uPAR (Figure 4D).

**Figure 5:**
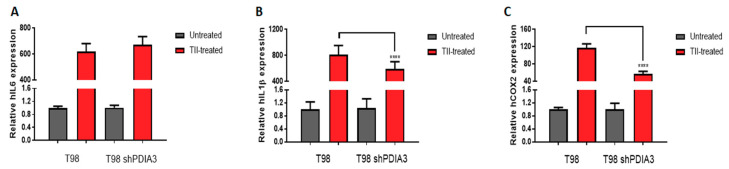
Effects of PDIA3 silencing on IL6, IL1β, and COX2 in activated human GB cells: T98G cells were treated or not with TII (TNFα, IL1β, and hIFNγ) for 8 h, and detection of transcripts was performed by qRT-PCR. Results indicate relative IL6 (**A**), IL1β (**B**), and COX2 (**C**) mRNA expression in TII-treated cells compared to untreated cells (control or silenced cells, to which the value of 1 was attributed) and are the mean ± SEM of three independent determinations. Statistically significant differences between TII-treated control andPDIA3-silenced cells are shown. **** *p* < 0.0001.

Moreover, in order to study the inflammatory response of glioma cells, we studied the effects of PDIA3 downregulation in T98G cells exposed 4 h stimulation with a pro-inflammatory cytokine mix (i.e., TNFα, IL1β, and hIFNγ, hereafter referred to as TII) followed by 3 washes and 24 h incubation with fresh media before collection of the culture supernatant (hereafter referred to as the pS-CM, condition that mimics a late stage of GB pathology. When control T98G cells were treated with TII for 8 h, the expression of IL6, COX2, and IL1β was increased of more than 600-fold, 115-fold, and 800-fold, respectively. Under the same experimental conditions, PDIA3-silenced cells showed a lower increment of COX2 (115-fold vs. 57-fold) and IL1β (800-fold vs. 590-fold) expression (Figure 5).

**Figure 6:**
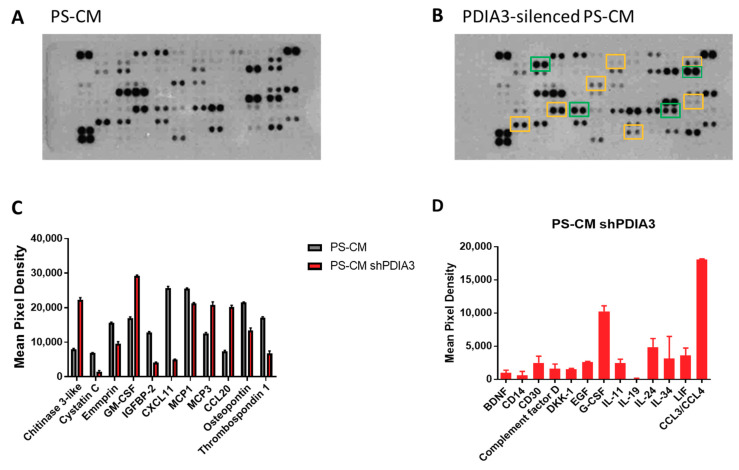
Semi-quantitative analysis of multiple cytokines, chemokines, growth factors, and other soluble proteins in cell culture media of pre-stimulated GB cells: (**A**,**B**) Results of protein analysis in PS-CM collected from control (**A**) and PDIA3-silenced (**B**) T98G cells. Yellow squares and green squares indicate the downregulated factors and upregulated factors, respectively. (**C**) Semi-quantitative analysis of the released proteins showing a statistically significant increase or decrease with *p* < 0.05. (**D**) Proteins whose expression was detected only after PDIA3 silencing. Data are mean ± SD (4). For a complete list of proteins that appear to be modified, see Supplementary Figure S3B.

In addition, supernatants were analyzed for the same panel of 105 cytokines, chemokines, growth factors, and soluble proteins released from both control and PDIA3 silenced-T98G cells (Figure 6A,B). In PS-CM collected from PDIA3-silenced cells, chitinase 3-like, granulocyte-macrophage colony-stimulating factor (GM-CSF), monocyte chemoattractant protein 3 (MCP3 or CCL7), and C-C motif chemokine ligand 20 (CCL20 or macrophage inflammatory protein 3—MIP3A) were significantly increased. On the contrary, cystatin C, emmprin, IGFBP2, monocyte chemoattractant protein 1 (MCP1 or CCL2), C-X-C motif chemokine 11 (CXCL11), osteopontin, and thrombospondin-1 were significantly decreased (Figure 6C). In addition, PDIA3-silenced T98G cells released cytokines and chemokines never produced from the control counterpart such as complement factor D, granulocyte colony-stimulating factor (G-CSF), and chemokine (C-C motif) ligand 3 and 4 (CCL3/CCL4) (Figure 6D).

**Figure 7:**
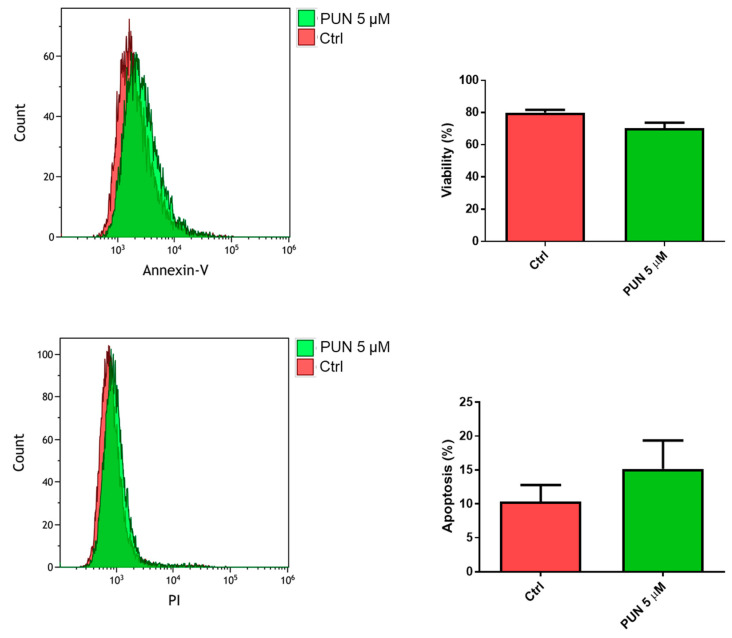
Viability of T98G cells after punicalagin (PUN) challenge: T98G cells were treated with 5 μM PUN for 24 h and then analyzed by the Annexin V-propidium iodide assay and flow cytometry. Viability was obtained from negative control cells without the Annexin V/PI conjugated fraction and expressed as percentage. Data are the mean (*n* = 8) ± SEM.

The influence of PDIA3 activity inhibition on glioma cell viability was tested after exposure of T98G cells to PUN by flow cytometry analysis with the annexin V-propidium iodide assay. After 24 h of treatment with 5 µM PUN (a concentration known to inhibit PDIA3 activity), viable T98G cells were more than 70%, with only 15% of cells in early apoptosis (Figure 7). Fifty µM PUN were instead highly toxic, inducing necrosis in 80% of the cell population.

### 2.3. Effects of PDIA3 Gene Silencing in Microglia-GB Interaction

#### 2.3.1. Effects of CMs from Control and PDIA3-Silenced T98G Cells on CHME-5 Viability and Activation

Based on previous data showing the toxicity of CMs of glioma cells on microglia [28], we tested the viability and activation status of microglial CHME-5 cells after exposure to B-CM and PS-CM collected from both control and PDIA3-silenced T98G cells.

**Figure 8:**
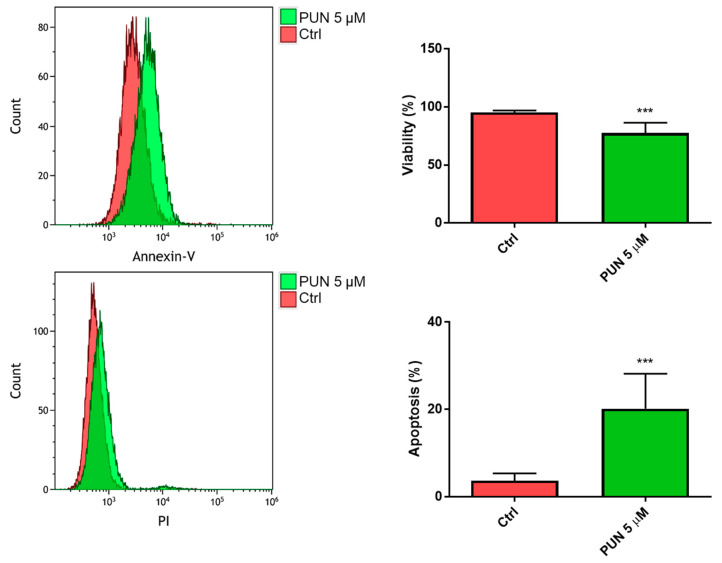
Viability of CHME-5 after punicalagin (PUN) challenge: CHME-5 cells were treated with 5 μM PUN for 24 h and then analyzed by the Annexin V-propidium iodide assay and flow cytometry. Viability was obtained from negative control cells without the Annexin V/PI conjugated fraction and expressed as percentage. *** *p* < 0.001. Data are the mean (*n* = 8) ± SEM.

When we analyzed the 24-h viability of CHME-5 cells after inhibition of PDIA3 activity elicited by 5 µM PUN, we found a significant reduction of viable cells with respect to untreated cells. In particular, we found more than 70% of viable cells and 20% of cells in the early apoptosis stage (Figure 8).

These data indicate that the PDIA3 pathway is important for microglial cell survival (tested by exposing CHME-5 cells to PUN). Conversely, downregulation of the same pathway on glioma cells does not affect microglial viability (tested using CMs obtained from PDIA3-silenced T98G cells on CHME-5 cells).

**Figure 9:**
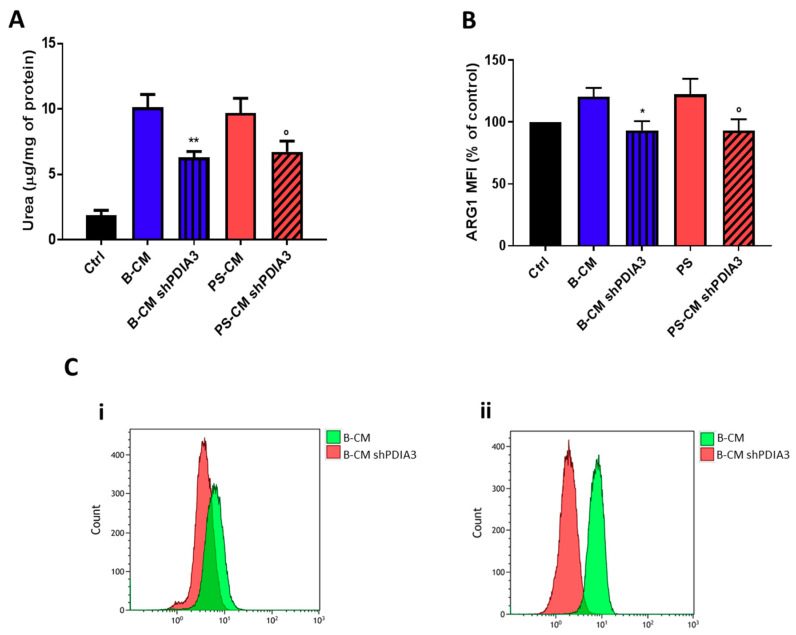
Analysis of M2 phenotype of CHME-5 cells exposed to GB-derived CM: (**A**) analysis of urea production by CHME-5 cells treated with the indicated CMs for 48 h and (**B**) flow cytometry analysis of ARG1 expression. CHME-5 cells were treated for 24 h with CMs from T98G and PDIA3-silenced T98G cells. Data are shown as percentage of control. (**C**) Flow cytometry analysis of ARG1: (**i**) fluorescence intensity measurements for B-CMs and (**ii**) fluorescence intensity measurements for PS-CMs. Data are shown as mean (*n* = 8) ± SEM. * *p* < 0.05; ° *p* < 0.05; ** *p* < 0.01.

With the purpose of evaluating the influence of PDIA3 expression in GB cells on microglia activation, ARG1 activity and expression (markers of M2 phenotype) in CHME-5 cells were measured. After 48 h exposure to CMs, urea release, an indicator of ARG1 activity, was reduced of 40% in CHME-5 cells treated with PDIA3-silenced T98G B-CM compared to cells exposed to control T98G B-CM. Similarly, CHME-5 treated with PS-CM obtained from PDIA3-silenced T98G showed a 40% decrease of urea compared to cells treated with PS-CM collected from T98G (Figure 9A). To confirm reduction of the M2 phenotype, we investigated the ARG1 expression on CHME-5 cells treated with B-CM and PS-CM from control T98G and PDIA3-silenced T98G cells. The results indicated a decrease of ARG1 expression in CHME-5 cells exposed to B-CM or PS-CM from PDIA3-silenced T98G cells compared to the same cells treated with CMs of control T98G cells (Figure 9B,C).

**Figure 10:**
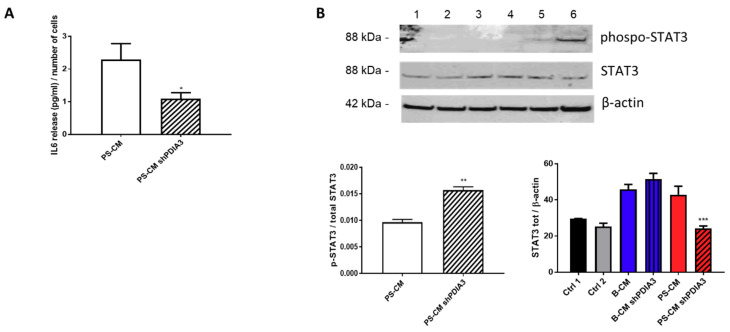
Effects of GB-derived CMs on the IL6-STAT3 pathway in microglial cells: (**A**) IL6 release, expressed as pg/mL per number of cells from CHME-5. Cells were treated with CMs for 24 h. IL6 was measurable only in cells treated with PS-CM. (**B**) Evaluation of p-STAT3 and STAT3 expression in microglia CHME-5 cells treated for 2 h with CMs from control and PDIA3-silenced T98G cells. Treatments: lanes 1 and 2, controls; lane 3, B-CM; lane 4, PDIA3-silenced B-CM; lane 5, PS-CM; and lane 6, PDIA3-silenced PS-CM. Data represent the results of densitometric analysis and are shown as the mean ± SEM of three independent determinations. * *p* < 0.05; ** *p* < 0.01; *** *p* < 0.001.

We also measured the release of the pro-inflammatory cytokine IL6 from CHME-5 cells treated with CMs. IL6 release was under the detection limit of the assay for all treatments except when the cells were stimulated with the PS-CMs. IL6 release induced by PS-CM obtained from control T98G cells was two-fold higher than that elicited by PS-CM deriving from silenced-PDIA3 T98G cells (Figure 10A). Moreover, we investigated the phosphorylation levels of signal transducer and activator of transcription 3 (STAT3), a transcription factor sensitive to cytokine levels. The results of western blot analysis indicated a significant increase of phosphorylated STAT3 in CHME-5 cells treated for 2 h with PS-CM from PDIA3-silenced T98G cells compared to cells exposed to PS-CM from control T98G (Figure 10B). On the contrary, total STAT3 tended to increase in CHME-5 cells treated with B-CM from PDIA3-silenced T98G cells compared to cells exposed to B-CM from control T98G, while it was significantly reduced in cells treated with PS-CM from PDIA3-silenced T98G compared to cells exposed to PS-CM from T98G (Figure 10B).

Therefore, all these data suggest that a partial blockade of PDIA3 in T98G cells might attenuate the pro-tumor microglia activation since it tends to reduce the M2 parameters (urea and ARG1) and IL6 release by microglia.

#### 2.3.2. Effects of PUN on Microglia Pro-Inflammatory Activation

In parallel, we also tested the effects of PDIA3 pharmacological inhibition on CHME-5 pro-inflammatory activation. Therefore, CHME-5 cells were treated for 24 or 48 h with TII alone or in combination with serial dilutions of PUN (1 nM–100 µM). Previously published studies indicate that PUN can interact with and inhibit the redox activity of PDIA3 in vitro at micromolar concentrations (IC50 lower than the micromolar, by a 0.8 µM) [25]. Moreover, concentrations between 10–20 µM of the inhibitor do not affect viability of a human neuroblastoma [25] or several human colon tumor cell lines [31].

**Figure 11:**
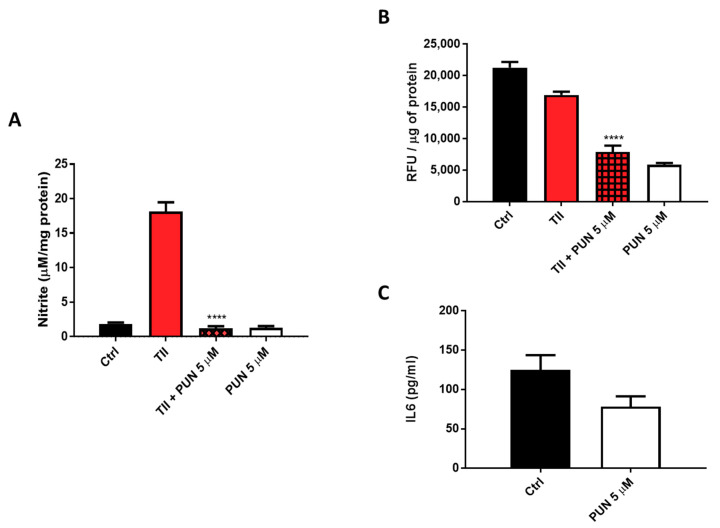
Anti-inflammatory effects induced by punicalagin (PUN) on CHME-5 cells: (**A**) CHME-5 cells were treated for 48 h with proinflammatory cytokines mix (TII) alone and/or in association with punicalagin. (**B**) Reactive oxygen species (ROS) production after 48 h of treatment with TII alone and in association with punicalagin, in Relative Fluorescence Units (RFU). (**C**) IL6 release after 24 h of treatment with punicalagin. Data are expressed as mean ± SEM. **** *p* < 0.0001. Data are the mean (*n* = 6) ± SEM.

Nitrites levels were significantly reduced when TII was combined with 5 µM PUN (Figure 11A and Figure S4). In addition to nitrite levels, evaluation of the intracellular production of total reactive oxygen species (ROS) was carried out, measuring them with the fluorescent dye, 2′,7′-dichlorodihydrofluorescein diacetate (H2DCF-DA) after treatment of CHME-5 cells for 48 h with TII alone and in association with 5 µM PUN. Data showed a significant reduction of fluorescence in cells exposed to PUN, alone or in combination with TII (Figure 11B), reinforcing the idea of multi-level anti-inflammatory activity. In addition, IL6 release in the culture media of CHME-5 cells was evaluated after 24 h of treatment with 5 µM PUN, and data were compared to IL6 release under basal conditions. In accordance with the observed reduction of IL6 release by PDIA3-silenced T98G and CHME-5 treated with PDIA3-silenced CMs, IL6 release was also reduced when cells were treated with 5 µM PUN compared to control (78.5 pg/mL versus 125 pg/mL on average) (Figure 11C). These data clearly indicated a strong correlation between IL6 and PDIA3.

**Figure 12:**
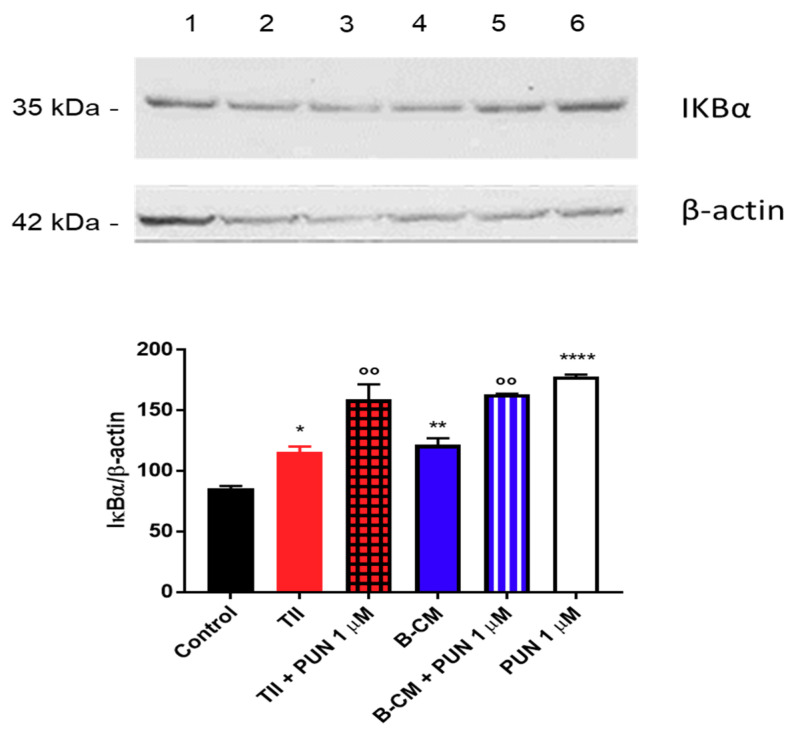
Evaluation of IκBα expression after punicalagin (PUN) challenge of microglial cells: CHME-5 cells were untreated (control) or treated with the different stimuli for 2 h. Treatments: lane 1, control; lane 2, TII; lane 3, TII + PUN 1 µM; lane 4, B-CM; lane 5, B-CM + PUN 1 µM; and lane 6, PUN 1 µM. TII, B-CM, and PUN are compared to the control; TII + PUN 1 µM and B-CM + PUN 1 µM are compared to the same treatments given alone. Data are shown as mean ± SD. * *p* < 0.05; **, °° *p* < 0.01; **** *p* < 0.0001.

To explain the anti-inflammatory effects of PUN on CHME-5 cells, we tested the involvement of the NF-κB pathway, and in particular, we measured IκBα levels (a NFκB inhibitor). Briefly, after 2 h treatment with PUN, a significant increment of IκBα was observed in cells treated with PUN alone or in combination with CMs (Figure 12). Taken together, these data confirm an involvement of PDIA3 in microglia activation and suggest an important anti-inflammatory activity of PUN in microglia cells.

## 3. Discussion

In this study, we reported, for the first time, the expression of PDIA3 in human GB specimens. In particular, we investigated the PDIA3 expression in the tumor and the nearby parenchyma from 18 GB patients and our data showed the expression of PDIA3 not only in tumor cells but also in GAMs, supporting its potential role in cellular and molecular processes related to GB. The involvement of PDIA3 in cancer progression was previously evaluated in other tumor types (i.e., renal cancer and hepatocellular carcinoma) [21,32]. In addition, in several cancer cell lines, the knockdown of PDIA3 affected different pathways involved in its physiological functions [14,33]. In this scenario, using a specific siRNA, we downregulated PDIA3 expression in the T98G human GB cell line. The effect of PDIA3 silencing on the expression of three genes known to be activated after a pro-inflammatory stimulus [28] in T98G cells was investigated: IL6, IL1β, and COX2. IL6 is known to regulate the inflammatory activation by decreasing pro-inflammatory cytokines and by upregulating anti-inflammatory cytokines [34,35]. Due to these properties, IL6 plays an important role in several cancers, including GB [35,36]. IL1β is a strong inducer of the inflammatory response that has been shown to prevent the metastatic colonization [37]. In addition, COX2 overexpression in cancer as well as its contribution to tumor development and progression have been extensively reported [38,39,40,41]. COX2 was found often overexpressed also in gliomas and correlated with tumor grade and shorter survival [42,43]. PDIA3-silenced T98G cells showed a downregulation of gene expression of COX2 when treated with activating stimuli, reinforcing the idea of the beneficial role of PDIA3 inhibition in glioma patients. Interestingly, the decrease of PDIA3 mRNA reduced the basal expression levels of IL6 and COX2 genes but increased IL1β expression, suggesting a pivotal role of PDIA3 on the inflammatory response of GB cells. Accordingly, when both PDIA3-silenced and control T98G cells to supernatants collected from GB cultures after cytokine stimulation (i.e., TII) were exposed for 4 h, we obtained a limited increase of IL1β and COX2, whereas IL6 was not modified or tended to be upregulated.

The present manuscript intends to mimic 2 different scenarios of GB pathology: an initial phase of the pathology, that we mimic culturing the T98G in basal conditions (B-CM), and a late phase, that we mimic by stimulating the T98G with pro-inflammatory cytokines (PS-CM). Both scenarios were also investigated after PDIA3 silencing. Using a microglia–glioma interaction cellular model, we investigated the involvement of PDIA3 in functional experiments mimicking the bidirectional interaction between GB (T98G cells) and microglia (CHME-5 cells). In this context, we evaluated the urea release, as a marker of M2 activation, from CHME-5 cells exposed to CMs collected from control or PDIA3-silenced T98G cells. In fact, ARG1 converts arginine to ornithine and urea, competing with nitric oxide synthase (NOS) (usually regarded as M1 marker), which utilizes arginine to produce nitric oxide [44,45]. Our previous data showed an increase of urea production when CHME-5 cells were treated with CMs from control T98G, reinforcing the M2 phenotype [28]. Here, we reported an attenuation of glioma-induced M2 phenotype when CHME-5 cells were exposed to CMs from PDIA3-silenced T98G cells. In fact, a reduced expression of ARG1 and a decrease of urea release were observed. Moreover, when microglial cells were treated with PDIA3-silenced T98G PS-CM, we found a decrease in IL6 levels. These data are in agreement with the previously described decrease of CCL2 and IL6 release by GAMs [46].

IL6 also triggers the JAK/STAT3 pathway, and in the GB pathology, high IL6 levels were associated with to poor outcome and overall survival [35,47]. In this regard, it should be noted that PDIA3 modulates STAT3 signaling [15,16] and that PDIA3 up- or downregulation has been related to cancer progression and inhibition of proliferation, respectively, through STAT3 [21,48]. Together with IL6, IL10 and FGF are also known to activate STAT3, which was found to be constitutively activated in glioma [49]. Moreover, STAT3 suppresses the antitumor immunity in GB [50]. In this scenario, we investigated STAT3 expression when microglial cells interact with control and PDIA3-silenced T98G cells. Interestingly, by comparing the effects of GB-derived CMs on microglial cells, we found opposite effects: on the one hand, total STAT3 was upregulated when cells were treated with PDIA3-silenced B-CM (probably due to upregulation of FGF-19 release); on the other hand, STAT3 was downregulated when cells were exposed to PDIA3-silenced PS-CM (probably due to downregulation of IL6 and FGF-19 release). Although we could not attribute the consequence of PDIA3 silencing to a direct effect on STAT3 activity, we hypothesized that PDIA3 might regulate the microglia phenotype through modulation of the STAT3 activation pathway. In addition, the different effects exerted by B-CM and PS-CM on STAT-3 might be explained with the fact that, in order to obtain PS-CM, cells were subjected to stimulation with cytokines that use STAT3-dependent pathways. Thus, also in this case, the stimulation of microglia cells can be affected by PDIA3 inhibition.

It is known that GAMs interact with glioma cells and that this crosstalk is supported by cytokines and chemokines released from both tumor cells and GAMs [51]. In particular, glioma cells recruit GAMs through the monocyte chemoattractant protein MCP-1 (or CCL2) [52]. Moreover, the macrophage colony-stimulating factor (M-CSF or CSF1), GM-CSF, and the stroma-derived factor (SDF-1 or CXCL12) are involved in the recruitment and M2 activation of GAMs [53,54,55]. In this context, we evaluated the expression of 105 cytokines, chemokines, growth factors, and other soluble proteins released from both control and PDIA3-silenced T98G cells. Several were the proteins released in B-CM, and some of them possessed proangiogenic activity, such as angiogenin, angiopoietin 2, and vascular endothelial growth factor-A (VEGF-A). Additionally, here, we reported the release of macrophage migration inhibitory factor (MIF), the inhibitory effect of which was likely mitigated by the release of MCP-1. More generally, T98G GB cells released chemoattractant and activation factors of the innate immune response. In PDIA3-silenced B-CM, we reported an enhancement of such factors (i.e., FGF-19; osteopontin, PDGF-AA, M-CSF, EGF, and G-CSF) together with a significant reduction of IFGBP-2 and IL8. More complex is the situation in PS-CM with 43 proteins released from glioma cells. In fact, PS-CM is a condition that mimics a late-stage pathology [27] and is characterized by the release of inflammatory cytokines, such as IL6, IL8, C-X-C motif chemokine 10 (CXCL10 or IP-10), as well as GM-CSF and MCP-1. Interestingly, when we evaluated the type of cytokines released in the PDIA3-silenced PS-CM, we observed a mitigation of the response triggered by activating stimuli with decreased secretion of most of the analyzed cytokines, such as IL17, IL8, and IL22. Some exceptions are chitinase 3-like, secreted by activated macrophages; GM-CSF, which is able to activate microglia; MCP-3, a chemotactic and activating factor of inflammatory cells; and CCL20, a chemotactic factor for lymphocytes. Moreover, PDIA3-silenced T98G PS-CM contained also IL24, G-CSF, LIF, and CCL3/CCL4 that were not present in PS-CM from control cells. In addition, it could be speculated that, when PDIA3 is silenced and glioma cells are exposed to an activating stimulus, glioma cells are no longer able to release high amounts of IL6 and IL8 to induce the M2 phenotype in GAMs. Accordingly, we detected the production of IL24, which is a tumor-suppressing protein, and LIF, which inhibits cell differentiation. Thus, PDIA3-silenced GB cells may continue to attract microglia/macrophages in the tumor microenvironment but tend to activate a different phenotype that might be speculated as tumor resolving. Hence, PDIA3 knockdown in GB cells provoked different responses in GAMs and its inhibition could exert beneficial therapeutic effects. Interestingly, PDIA3 can be also pharmacologically inhibited through the administration of PUN, a natural noncompetitive inhibitor [25] found to reduce neuroinflammation in microglia [56,57,58]. However, its activity in gliomas is poorly characterized and seems to be related to induction of autophagic cell death and apoptosis [59]. In this context, we found that PUN exerted anti-inflammatory effects on microglial cells.

## 4. Materials and Methods

### 4.1. Materials

Cell culture reagents (Dulbecco’s modified Eagle’s medium (DMEM) and Fetal calf serum (FCS)) were from Invitrogen Corporation (Paisley, Scotland). Antibiotics were from Biochrom AG (Berlin, Germany). Puromycin was from VWR International (Cat. No.: J593, VWR, Milan, Italy), and PUN was from Merck (Darmstadt, Germany). PUN was dissolved in water. The human recombinant interleukin-1β (IL1β), human interferon γ (IFNγ) and recombinant human Tumor necrosis factor α (TNFα) were purchased from R&D System (Minneapolis, MN, USA). The following antibodies were used: mouse monoclonal anti-β-actin (Cat. No. A5316; clone AC-74; Merck, Darmstadt, Germany), rabbit monoclonal anti-phospho-STAT3 (Cat. No. 9145 Tyr705; Cell Signaling Technology, Danvers, MA, USA), mouse monoclonal anti-STAT3 (Cat. No. 9139T 124H6; Cell Signaling Technology, Danvers, MA, USA), goat polyclonal anti-AIF-1/IBA1 (Cat. No. NB100-1028 Novus Biologicals, Centennial, CO, USA), rabbit polyclonal anti-IκBα (Cat. No. sc-371 Santa Cruz Biotechnology, Dallas, TX, USA), rabbit polyclonal anti-4EBP1 (Cat. No.: A300-501A; Bethyl Laboratories, Montgomery, TX, USA), rabbit polyclonal anti-ERp57/PDIA3 (kindly provided by Fabio Altieri), goat anti-rabbit IgG peroxidase conjugated (Cat. No.: 111-035-045, Jackson ImmunoResearch Laboratories, West Grove, PA, USA), and goat-anti-mouse IgG peroxidase conjugated (Cat. No.: A3682, Merck, Darmstadt, Germany).

### 4.2. Patients and Specimens

We studied 18 GB cases from the previously described 42 adult patients [26] who underwent surgery for primary GB at the Neurosurgery Department, “Fondazione Policlinico Gemelli” (Rome, Italy), from March 2005 to September 2011. Diagnosis of GB was established on histological examination according to the WHO classification (grade IV) of tumors of the CNS. In all cases, total tumor removal was achieved, allowing us to obtain tissues samples from both the tumor and the surrounding macroscopic normal brain tissue, here defined as “periphery” (1–2 cm from one site tumor border; larger resections were performed in tumors that grew far from eloquent areas). The demographic characteristics of patients are reported in Table 1. All patients provided written consent to use their specimens for research studies, and the research proposal was approved by the ethics committee of hospital A. Gemelli (Prot. A/205/2011) [26].

### 4.3. Tissue Preparation

Human tumor tissues obtained by surgical GB resection were fixed in 4% paraformaldehyde in 0.1 M phosphate buffered saline (PBS, pH 7.6) overnight at 4 °C. Dehydration of tissues was performed through a graded ethanol series (80% and 95% for 1 h each, followed by 100% ethanol overnight). Two 100% xylene washes were done for 1 h each and then 1 h in 60 °C Paraplast Plus (Tyco/Healthcare, Mansfield, MA, USA). After a change of Paraplast Plus, tissues were incubated in a 60 °C vacuum oven for 2 h prior to placing in molds to cool and solidify. Three μm-thick sections were cut and collected on the superfrost plus slides (ThermoFisher Scientific, MA, USA).

### 4.4. Immunohistochemistry

The PT Link (Dako, Agilent Technologies, Santa Clara, CA, USA) was used to deparaffinize and rehydrate the sections and to unmask antigen sites. Slides were immersed in 10 mM citrate buffer, pH 6.0, for 10 min at 97 °C and then cooled and washed in TBS (tris buffered saline, ThermoFisher Scientific, MA, USA). Endogenous peroxidase activity was inhibited by incubating the slides with Peroxide Block (ScyTek Laboratories, Logan, UT, USA) for 7 min. After washing in distilled water and then in TBS, sections were treated with Avidin-Biotin Kit (Biocare-Medical Pacheco, Pacheco, CA, USA) to reduce background staining caused by endogenous biotin. After 3 washes in TBS, nonspecific binding was blocked by 10 min incubation with Background Punisher (Biocare-Medical, Pacheco, CA, USA). Sections were incubated overnight at 4 °C with goat anti-Human IBA1 polyclonal antibody (1:250, Novus Biologicals, Centennial, CO, USA), washed extensively in TBS, and subsequently incubated with Ultratek HRP kit (ScyTek Laboratories, Logan, UT, USA), and the reaction was revealed by incubation with 3,3′-diaminobenzidine (Biocare-Medical, Pacheco, CA, USA). The same slides were washed extensively in distilled water and TBS and blocked with Background Punisher (Biocare-Medical, Pacheco, CA, USA). The rabbit anti-PDIA3 antibody (1:500, Sigma-Aldrich) was incubated overnight at 4 °C and, after 3 washes in TBS, slides were incubated with the MACH 2 Rabbit HRP-Polymer (Biocare-Medical, Pacheco, CA, USA). The PDIA3-associated signal was revealed by incubation with Vina Green chromogen kit (Biocare-Medical, Pacheco, CA, USA).

For quantitative analysis, two blinded examiners counted the number of PDIA3+, IBA1+, or PDIA3+ and IBA1+ co-stained cells in a total of 50 cells in three randomly different areas of the slides [60]. The average of six counts was reported as percentage.

### 4.5. Cell Cultures

The human microglia cell line (CHME-5; RRID: CVCL_5J53) was kindly provided by Pierre Talbot (INRS-Institut Armand-Frappier, Laboratoire de Neuroimmunovirologie, Québec, Canada) [61]. CHME5 cells were grown in DMEM media containing 10% FCS and antibiotics; experimental conditions were reached with DMEM at low concentration of FCS (1%), and cells were split at 80% of the confluence.

The human GB cell line T98G (ATCC^®^ CRL-1690™) was obtained from the American Type Culture Collection (ATCC, Manassas, VA, USA). T98G cells were grown in DMEM containing 10% FCS and antibiotics. Experimental conditions were conducted with DMEM containing 1% FCS and antibiotics for 24 h and 48 h. This cell line was authenticated by STR profiling (BMR genomics, Padova, Italy). Conditioned media (CM) from activated GB cells was generated according to a previously described protocol [28]. Briefly, basal conditioned media (B-CM) was prepared by incubating cells in plain medium for 4 h, followed by 3 washes with PBS and addition of fresh plain medium for 24 h. Pre-stimulated conditioned media (PS-CM) was prepared by incubating cells for 4 h with a mixture of cytokines (10 ng/mL TNFα, 10 ng/mL IL1β, and 10 UI/mL hIFNγ, referred to as TII), followed by 3 washes with PBS and addition of fresh plain medium for 24 h. B-CM and PS-CM were collected, centrifuged to remove cellular debris, and stored at –80 °C.

### 4.6. PDIA3 Short Interfering RNA (siRNA) Transfection

The day before transfection, cells were seeded in a 6-well plate (5 × 10^5^/well) and grown in normal conditions. Transfection was carried out by using Lipofectamine™ 2000 (Invitrogen, Carlsbad, CA, USA) and the 7052 bp plasmid pLKO.1-puro Vector (ShRNA–PDIA3 human) (Sigma-Aldrich, St. Louis, MO, USA, kindly provided by Fabio Altieri, Sapienza University, Rome, Italy) at 1 µg/mL final concentration. Cell lines were incubated 6 h with the transfection complex, and after 48 h incubation, antibiotic selection was performed by adding 1 µg/mL puromycin. Cells were grown with puromycin for at least two weeks before analyzing PDIA3 gene and protein expression.

### 4.7. Nitrite Assay

Inducible nitric oxide synthase (iNOS) activity was assessed indirectly by measuring nitrite accumulation in the incubation media. Briefly, an aliquot of the cell culture media (80 μL) was mixed with 40 μL Griess Reagent (Merck, Darmstadt, Germany) and the absorbance was measured at 550 nm in a spectrophotometric microplate reader (PerkinElmer, Waltham, MA, USA). A standard curve was generated during each assay in the range of 0–100 μM concentrations using NaNO_2_ (Merck, Darmstadt, Germany) as the standard. In this range, standard detection was linear and the minimum detectable concentration of NaNO_2_ was 3.12 μM. In the absence of stimuli, basal levels of nitrites were below the detection limit of the assay at all the time points analyzed. The NO levels were normalized with the protein content determined by Bradford method (Bio-Rad Laboratories, Hercules, CA, USA) using bovine serum albumin (BSA) as the standard.

### 4.8. Urea Assay

Urea levels in CHME5 and T98G cells were detected by the QuantiChrom Urea Assay kit (BIOassay System, Hayward, CA, USA), used according to the manufacturer’s instructions. Briefly, after 48 h of incubation with the B-CM and PS-CM, an aliquot of cell culture media (50 µL) was mixed with 200 µL Urea Reagent (Bioassay system, Hayward, CA, USA) and the absorbance measured at 430 nm in a spectrophotometric microplate reader (PerkinElmer, Waltham, MA, USA). A standard curve was generated during each assay in the range of concentrations 0–100 µg/mL using urea as standard. In this range, standard detection was linear and the minimum detectable concentration of urea was 3.12 µg/mL. The protein content in each sample was determined by Bradford method (Bio-Rad Laboratories, Hercules, CA, USA) using BSA as the standard.

### 4.9. Flow Cytometry Analysis

For analysis of intracellular proteins, cells were fixed and permeabilized with Fix/Perm buffer (ThermoFisher Scientific, Waltham, MA, USA) and then incubated with primary monoclonal antibody anti-ARG1 (C-2) (Santa Cruz Biotechnology, Dallas, TX, USA) and goat anti-mouse Alexa Fluor^®^-488 (ThermoFisher Scientific, Waltham, MA, USA) secondary monoclonal antibody.

### 4.10. Cell Viability and Toxicity

For viability and apoptosis analysis, cells were incubated with propidium iodide and annexin V-FITC kit (NBP2-29373, Novus Biological, Centennial, CO, USA). The assay was conducted following the manufacturer’s instructions. Compensation control cells were included in the kit and unstained cells were used as a negative control. Flow cytometry analysis was conducted with FC 500 (Beckman Coulter, Brea, CA, USA), and the data were analyzed with Kaluza software (Beckman Coulter, Brea, CA, USA). At least 50,000 events were acquired.

Cell viability and mortality were also measured using the CellTiter-Glo^®^ Luminescent Cell Viability Assay (Promega, Madison, WI, USA) and the RealTime-Glo™ MT Cell Viability Assay (Promega, Madison, WI, USA), respectively, according to the manufacturer’s instructions.

### 4.11. Reactive Oxygen Species (ROS) Assay

Detection of intracellular ROS was performed using H2DCF-DA (2,7-dichlorodihydrofluorescein diacetate) as a probe. Briefly, cells were treated with TII or with TII + PUN for 48 h and, then, after replacing the incubation medium with Balanced Salt Solution (BSS—NaCl 124 mM, KCl 5.8 mM, dextrose 10 mM, Hepes 20 mM, and CaCl_2_(H_2_O)2 0.3 mM), incubated for 30 min at 37 °C. Afterwards, H2DCF-DA (20 Μm) was added to the culture and cells were incubated for 45 min at 37 °C. The fluorescence signal was quantified using a microplate fluorescence reader (VictorXTM4 microplate reader, PerkinElmer, Waltham, MA, USA), using 485 nm as the excitation and 535 nm as the emission wavelengths.

### 4.12. Real-Time Quantitative RT-PCR (qRT-PCR) Analysis

Total RNA from cell lines was extracted using the TRIzol reagent protocol following the manufacturer’s instructions. RNA concentration was measured using the Qubit™ RNA HS Assay Kit (Thermo Fisher Scientific). Aliquots (1 µg) of RNA were converted to cDNA using random hexamer primers. Quantitative changes in mRNA levels were estimated by qRT-PCR using the following conditions: 35 cycles of denaturation at 95 °C for 20 s, annealing and extension at 60 °C for 20 s, and using the Brilliant III Ultra-Fast SYBR^®^ Green QPCR Master Mix (Stratagene, San Diego, CA, USA). PCR reactions were carried out in a 20-µL reaction volume in AriaMX real-time PCR machine (Agilent, Santa Clara, CA, USA). Gene expression was evaluated using the following primers: human PDIA3, forward GCCACAGTCTTGTCCTCAAACTTG and reverse TTCCTAAAAGCAGCCAGCAACTTG; human Cyclooxygenase-2 (COX2), forward TTGCTGGCAGGGTTGCTGGTGGTA and reverse CATCTGCCTGCTCTGGTCAATCGAA3; human IL6, forward GGCTCATTCTGCCCTCGAGCC and reverse: GGACCGAAGGCGCTTGTGGAG; human IL1β, forward AGCCATGGCAGAAGTACCGT and reverse TCCATGGCCACAACAACTGA; and human Glyceraldehyde 3-phosphate dehydrogenase (GAPDH), forward CCCTCGCCATGGTAAATACAT and reverse ACTGGATGGTACGCTTGGTCT. Relative mRNA concentrations were calculated from the take-off point of reactions (threshold cycle, Ct) using the comparative quantitation method provided by AriaMX software (Agilent Aria v1.5) and based upon the −ΔΔCt method. Ct values for GAPDH expression served as a normalizing signal.

### 4.13. IL6 Quantification and Multiple Cytokines Analysis

IL6 levels in the culture media were detected using a specific enzyme-linked immunosorbent assay (ELISA, R&D System, Minneapolis, MN, USA), and levels of other cytokines and chemokines were measured using the Proteome Profiler Human Cytokines XL kit (R&D System, Minneapolis, MN, USA). The assays were conducted following the manufacturer’s instructions. Five hundred µL of cell culture supernatant were run on each assay. Each spot was measured with image analysis software, and the average pixel density of negative control spots was taken as background and subtracted. Each dataset was represented with GraphPad Software Prism v.7.04 (GraphPad Software, San Diego, CA, USA).

### 4.14. Immunoblot Analysis

Cells were lysed in RIPA buffer (1 mM EDTA, 150 mM NaCl, 1% igepal, 0.1% sodium dodecyl sulfate, SDS, 0.5% sodium deoxycholate, 50 mM Tris–HCl, and pH 8.0) (Merck, Darmstadt, Germany) containing protease inhibitor cocktail diluted 1:250 (Merck, Darmstadt, Germany). The protein content in each sample was determined by Bradford method (Bio-Rad, Hercules, CA, USA) using BSA as a standard. A 100-µg aliquot of protein was mixed with 4× Bolt™ LDS Sample Buffer (Cat. No.: B0008, Invitrogen, Carlsbad, CA, USA) and 10× Bolt™ Sample Reducing Agent (Cat. No.: B0009, Invitrogen, Carlsbad, CA, USA), boiled for 5 min, and separated through 4–12% bis-tris plus gel (Invitrogen, Carlsbad, CA, USA). After electrophoresis, proteins were transferred to nitrocellulose membranes by iBlot™ 2 Gel Transfer Device (Invitrogen, Carlsbad, CA, USA). The membranes were incubated in the presence of the primary and secondary antibodies in the iBind™ Flex Western Device (Cat. No.: SLF2000, Invitrogen, Carlsbad, CA, USA) for β-actin. Primary antibodies for IκBα, phospho-STAT3, and total STAT3 (1:1000) were incubated overnight with gentle shaking at 4 °C. After removal of primary antibodies, membranes were washed 3 times in Flex Solution and further incubated for 1 h at room temperature in the presence of specific secondary antibodies (1:15,000 and 1:3000 for anti-rabbit and anti-mouse antibodies, respectively). Following three washes in Flex Solution, bands were visualized by incubation in ECL reagents (ThermoFisher Scientific, Waltham, MA, USA) and exposure to Hyperfilm ECL (GE Healthcare, Chicago, IL, USA).

### 4.15. Statistical Analyses

Data were described as median ± Standard Deviation (SD) or SEM as indicated in figure legends. Statistical analysis of the differences between pairs of groups was performed by Student’s *t* test. For multiple comparisons, ANOVA analysis followed by Sidak’s posttest was used. Statistical significance was determined at the α = 0.05 level. Differences were considered statistically significant when *p* < 0.05. Statistical analysis was performed with GraphPad software Prism version 7.04 (GraphPad Software, San Diego, CA, USA). Different symbols were used to indicate statistical differences, in particular, * *p* < 0.05, ** *p* < 0.01, *** *p* < 0.001 and **** *p* < 0.0001.

## 5. Conclusions

We reported that ERp57/PDIA3 is expressed in specimens from GB patients, both in GB cells and GAMs. In GB cells, downregulation of ERp57/PDIA3 resulted in alterations of the pattern of cytokines secreted by tumor cells that affect the surrounding environment in their favor. In particular, a reduced GAM polarization towards the M2 phenotype was observed (as indicated by the lower expression and activity of the M2 marker ARG1). Silencing of PDIA3 in GB cells resulted in reduced release by tumor cells of IL6 and COX2 and increased production of IL1β. Altogether, these alterations in the pattern of cytokine secretion might contribute to creating an antitumor microenvironment. Furthermore, treatment of microglial cells with a specific ERp57/PDIA3 inhibitor, PUN increased apoptosis and reduced their pro-inflammatory activity (lower nitrites, ROS, and IL6 production). Thus, ERp57/PDIA3 might represent a suitable strategy to be further evaluated for GB treatment in preclinical in vivo studies.

## Figures and Tables

**Table 1:** Demographic characteristics of GB patients.

Study Cohort (18 Patients)	Demographic Characteristic	Number of Patients (N)	% of Patients
Age	over 70	5	27.8
	60–70	5	27.8
	50–60	3	16.7
	40–50	3	16.7
	under 40	1	5.6
	NA	1	5.6
Gender	Male	13	72.2
	Female	5	27.8
WHO grade classes	IV	18	100
Tumor	Primary	14	77.8
	Recurrent	4	22.2
Tumor location	Frontal	6	33.3
	Occipital	2	11.1
	Temporal	5	27.8
	Parietal	1	5.6
	Tempo-Parietal	1	5.6
	NA	3	16.6

NA: not available.

## Supplementary Materials

The following are available online at https://www.mdpi.com/1422-0067/21/21/8214/s1, Figure S1: PDIA3/IBA1 double staining in GBM specimens from 18 patients, Figure S2: PDIA3 gene expression and silencing in GB cells, Figure S3: Detail of all proteins that are modified in conditioned media, Figure S4: Reduction of nitrite levels elicited by punicalagin on CHME-5 cells.

## Abbreviations

PDIA3	Protein Disulfide Isomerases 3
GB	Glioblastoma
GAMs	Glioma Associated Microglia/macrophages
ER	Endoplasmic Reticulum
ERp57	Endoplasmic Reticulum Protein 57
PUN	Punicalagin
TCGA	The Cancer Genome Atlas
CM	Conditioned Medium
IL6	Interleukin-6
ERp60	Endoplasmic Reticulum Protein 60
PDI	Protein Disulfide Isomerase
TRX	Thioredoxin Domain
NFκB	Nuclear Factor kappa-light-chain-enhancer of activated B cells
mTOR	Mammalian Target of Rapamycin
STAT3	Signal Transducer and Activator of Transcription 3
CCR5	C-C chemokine Receptor type 5
ROS	Reactive Oxygen Species
TNFα	Tumor Necrosis Factor α
siRNA	small interfering RNA
COX2	Cyclooxygenase-2
IL1β	Interleuckin-1 β
IFNγ	Interferon gamma
B-CM	Basal Conditioned-Medium
PS-CM	Prestimulated Conditioned-Medium
FGF-19	Fibroblast Growth Factor 19
PDGF-AA	Platelet-Derived Growth Factor A-A
IGFBP2	Insulin-like Growth Factor-Binding Protein 2
IL8	Interleukin-8
DKK-1	Dickkopf-related protein 1
M-CSF	Macrophage Colony-Stimulating Factor
uPAR	Urokinase Receptor
GM-CSF	Granulocyte-Macrophage Colony-Stimulating Factor
MCP-3	Monocyte-Chemotactic Protein 3
CCL20	C-C Chemokine ligand 20
MCP-1	Monocyte-Chemotactic Protein 1
CXCL11	C-X-C motif Chemokine 11
G-CSF	Granulocyte Colony-Stimulating Factor
CCL3/CCL4	C-C Chemokine ligand 3 and 4
ARG1	Arginase-1
NO	Nitric Oxide
IκBα	Nuclear factor of kappa light polypeptide gene enhancer in B-cells inhibitor, alpha
NOS	Nitric Oxide Synthases
CCL2	C-C Chemokine ligand 2
IL10	Interleukin-10
SDF-1	Stromal cell-Derived Factor 1
VEGF-A	Vascular Endothelial Growth Factor-A
MIF	Macrophage migration Inhibitory Factor
IP-10	Interferon gamma-induced Protein 10
IL24	Interleukin-24
