# Supplementary material for: PDIA3 Expression in Glioblastoma Modulates Macrophage/Microglia Pro-Tumor Activation
Source: Int J Mol Sci. 2020 Nov 3;21(21):8214. doi: 10.3390/ijms21218214 (PMC7662700; doi:10.3390/ijms21218214)
Supplement: Supplementary file 1 [file ijms-21-08214-s001.zip › ijms-966417-revised-supplementary/Figure S1 new.docx]

**Additional file 1**

**Figure S1**

**#1 patient**


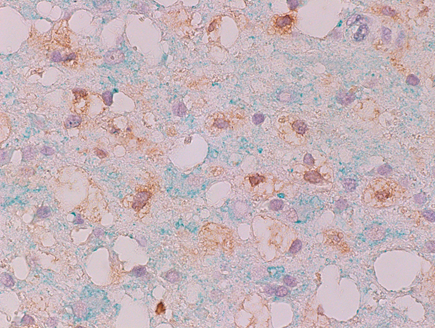

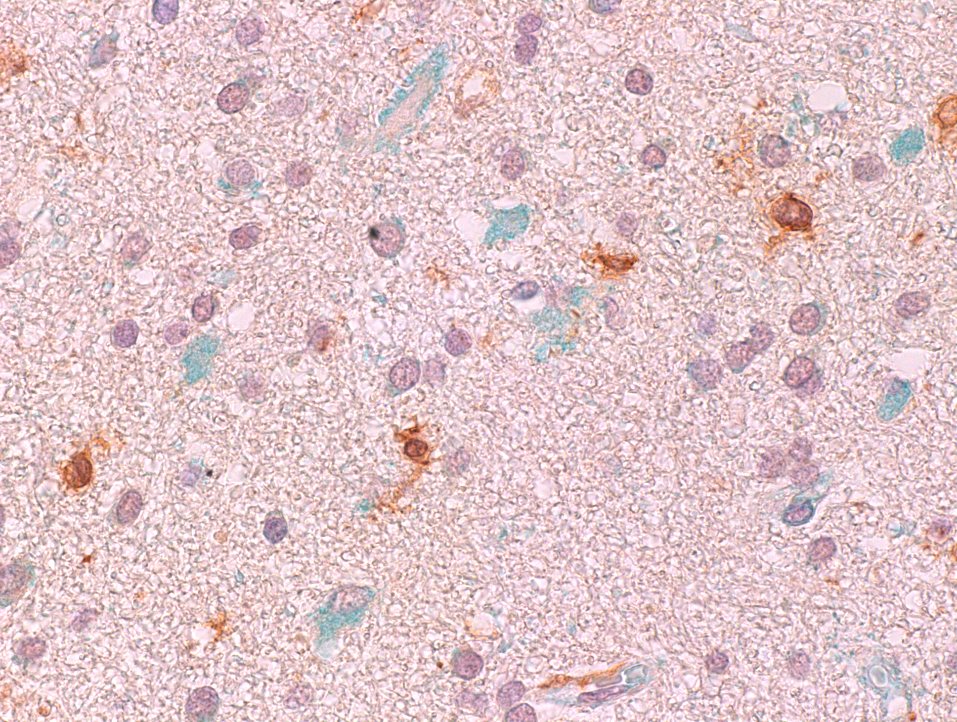


**#2 patient**


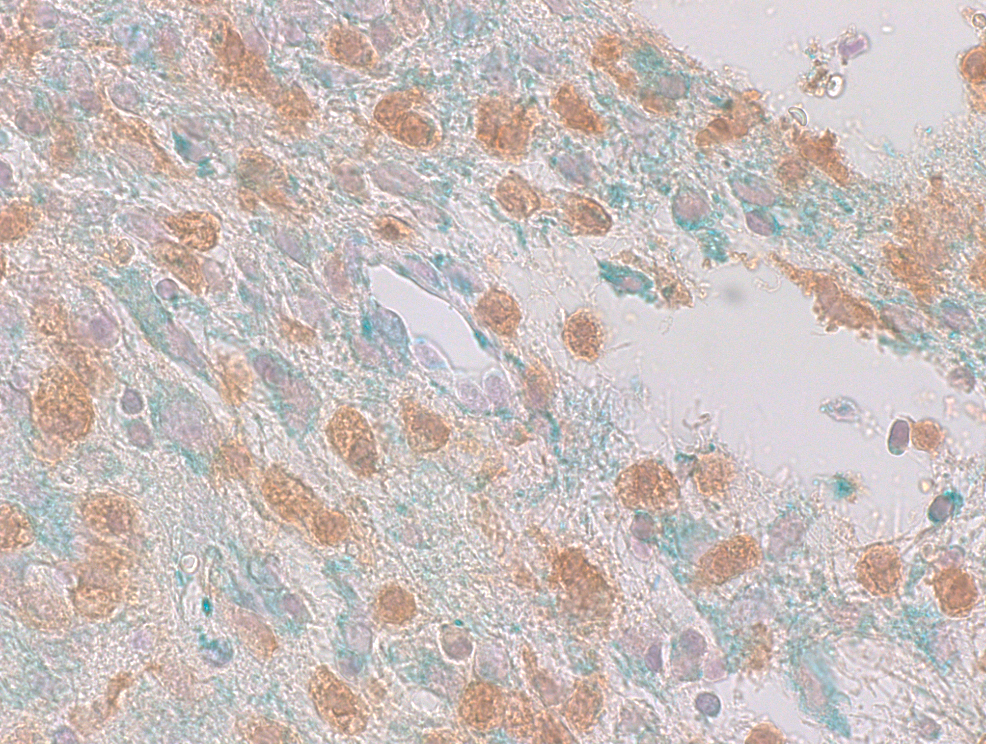

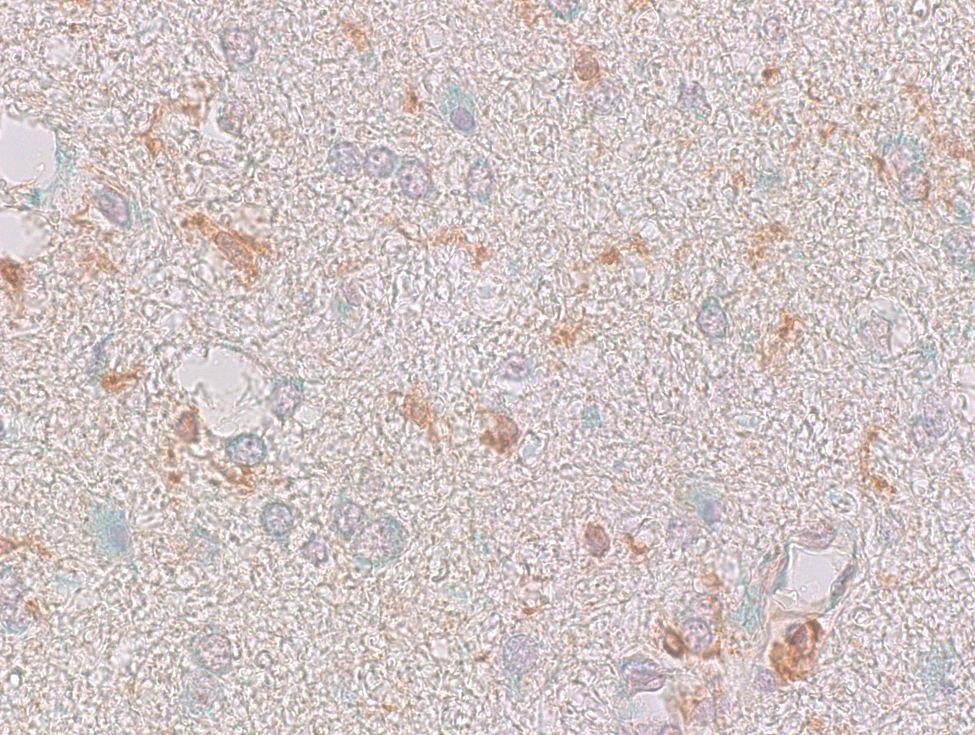


**#3 patient**


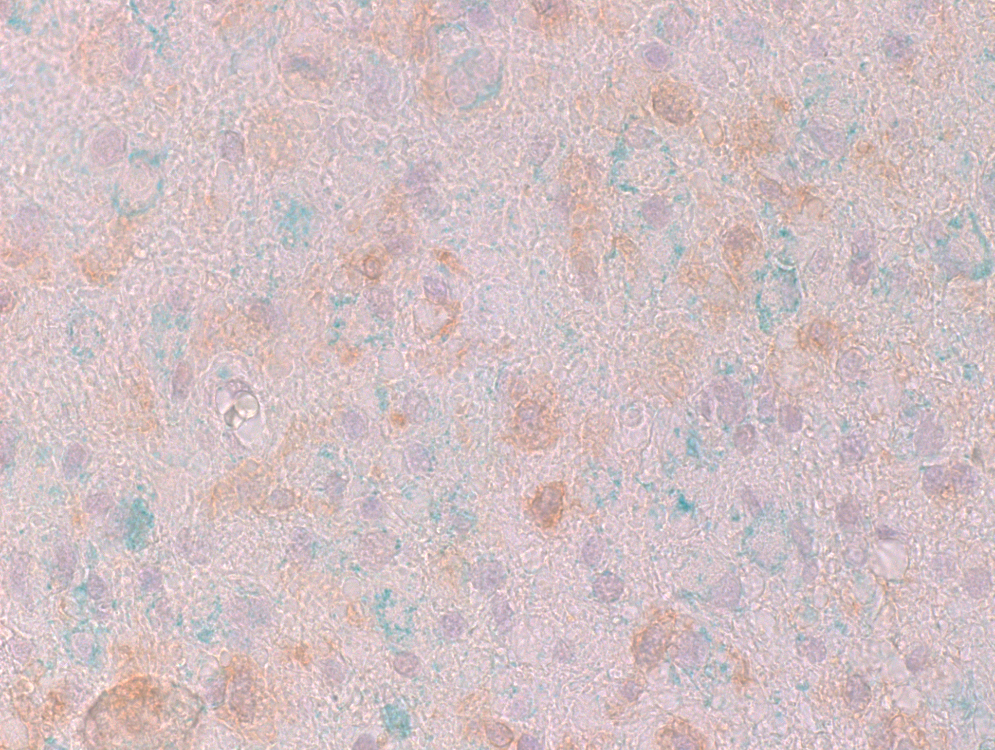

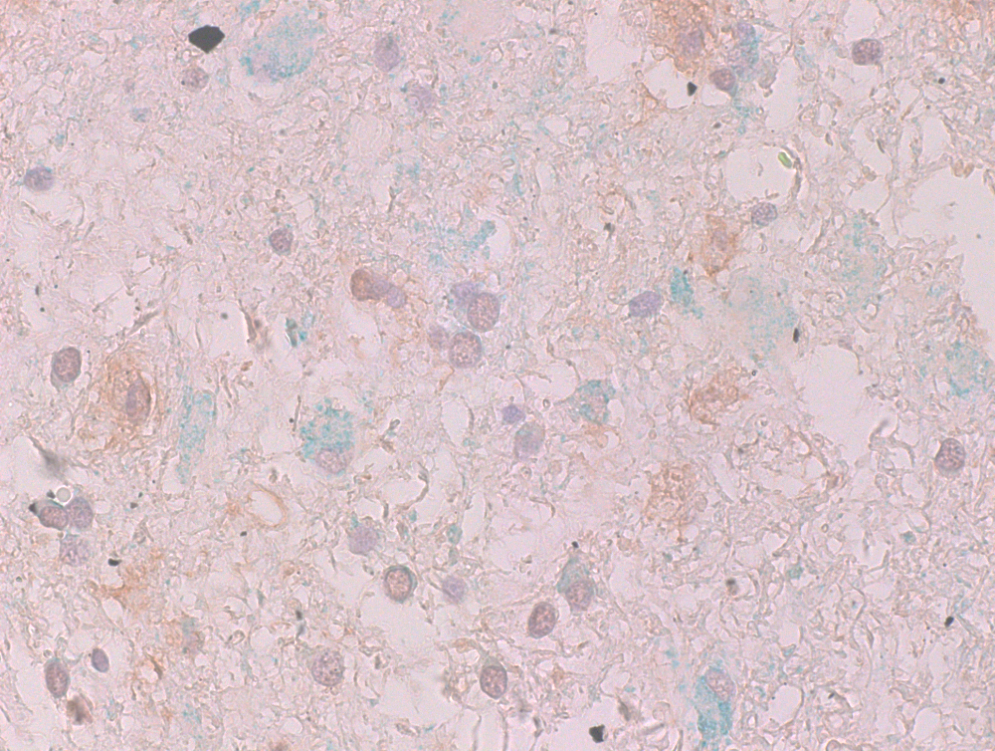


**#4 patient**


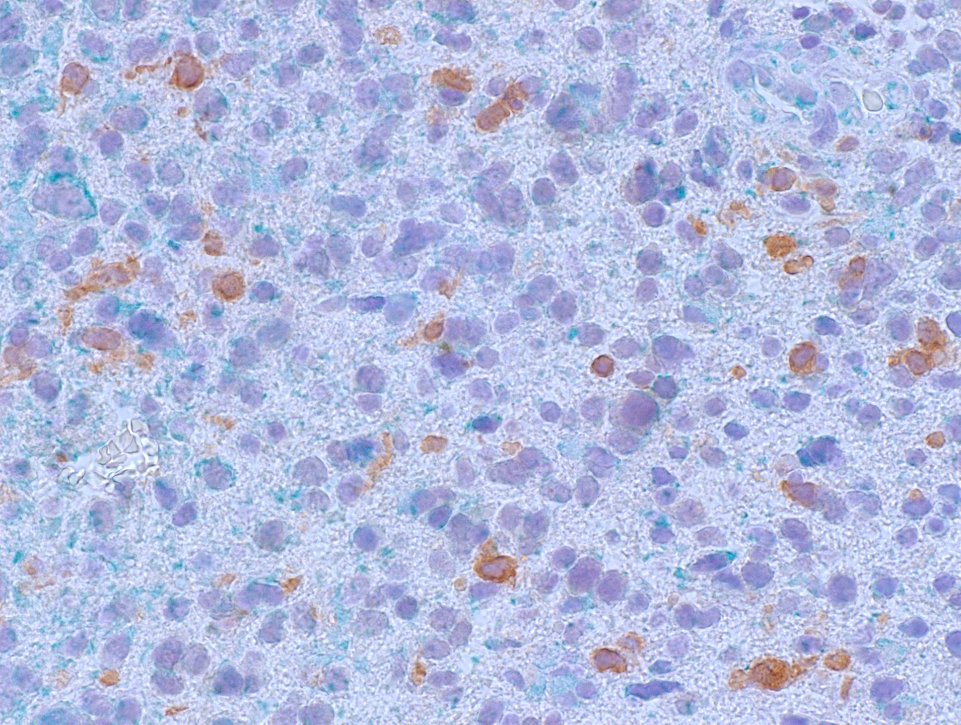

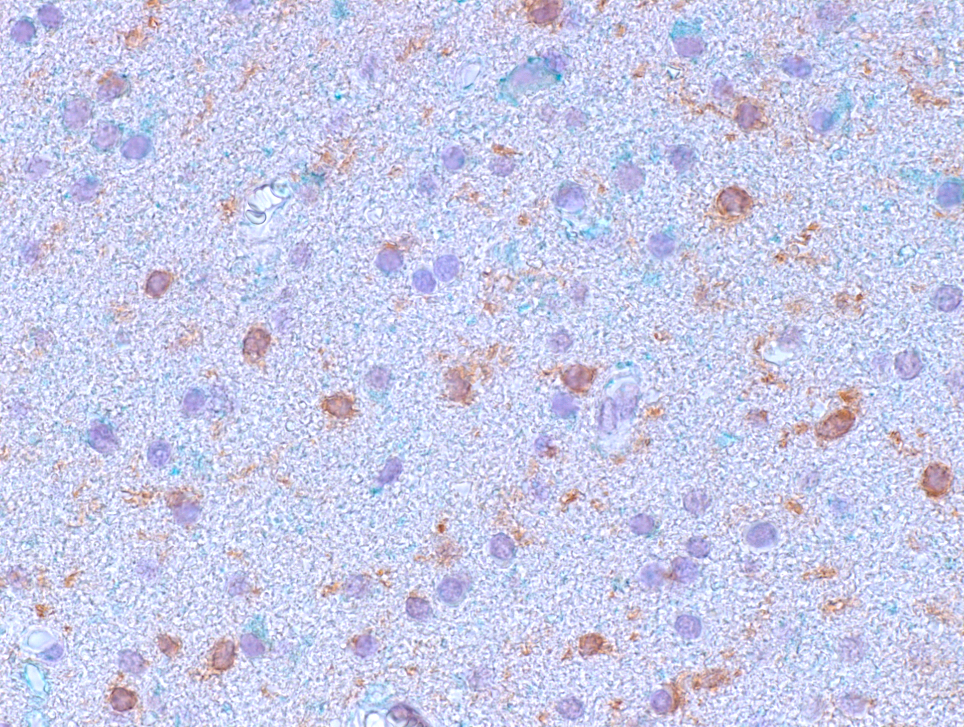


**#5 patient**


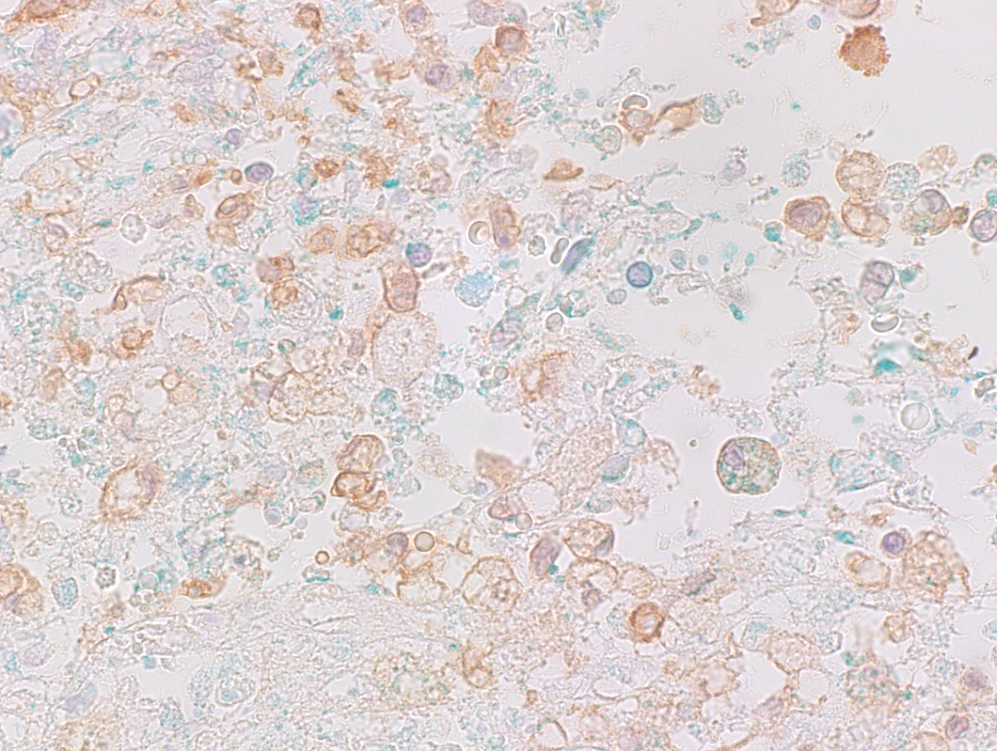

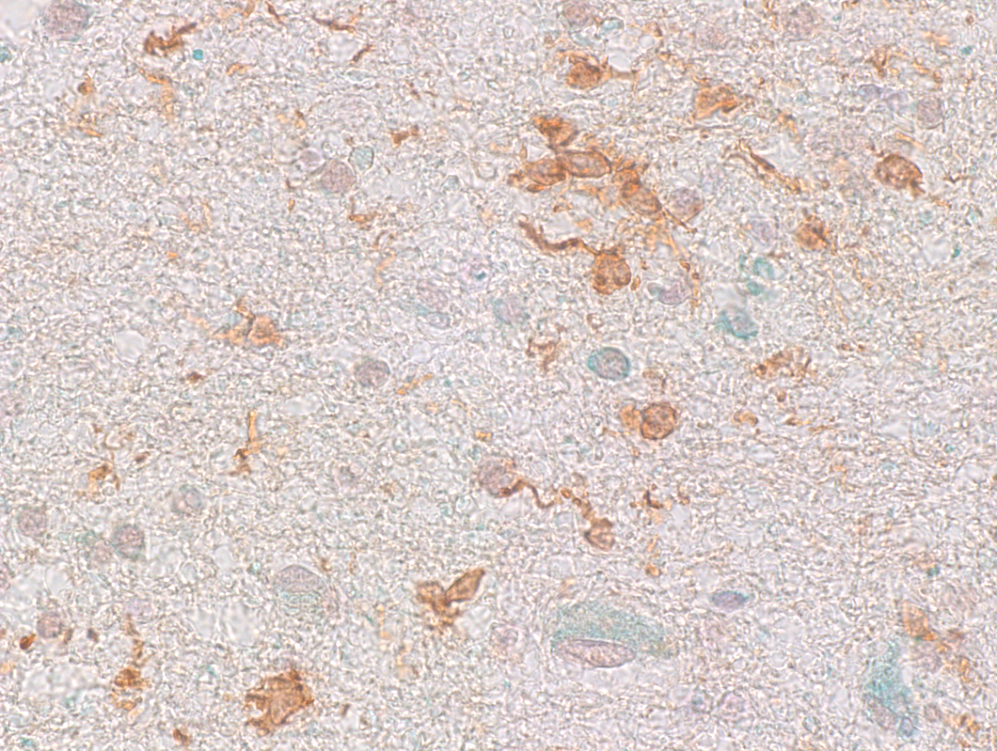


**#6 patient**


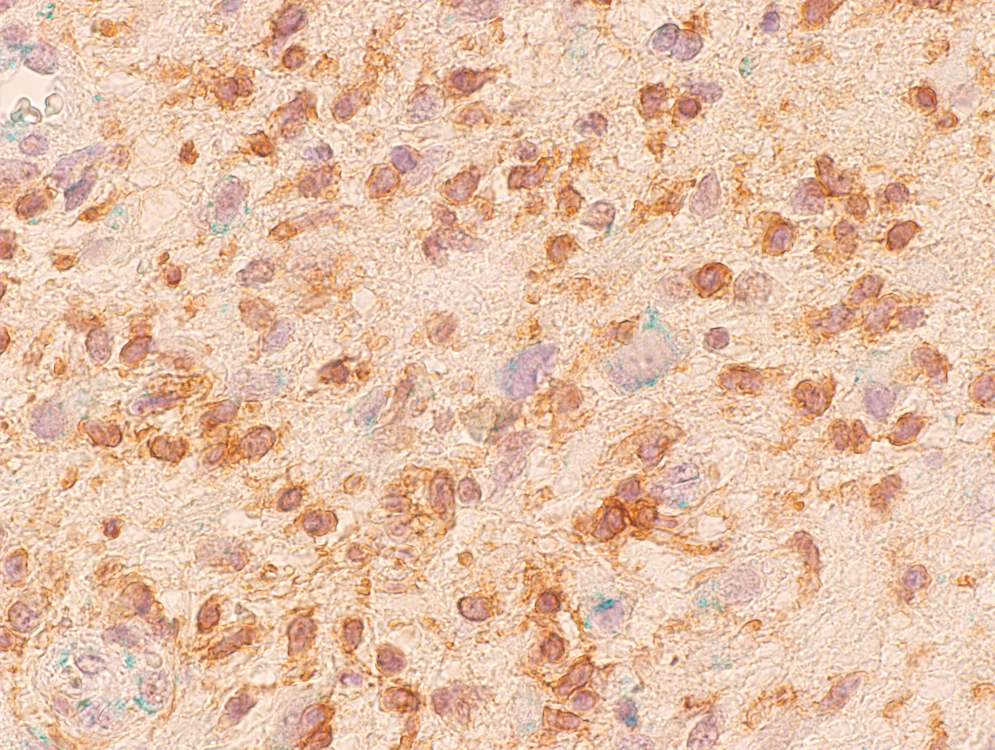

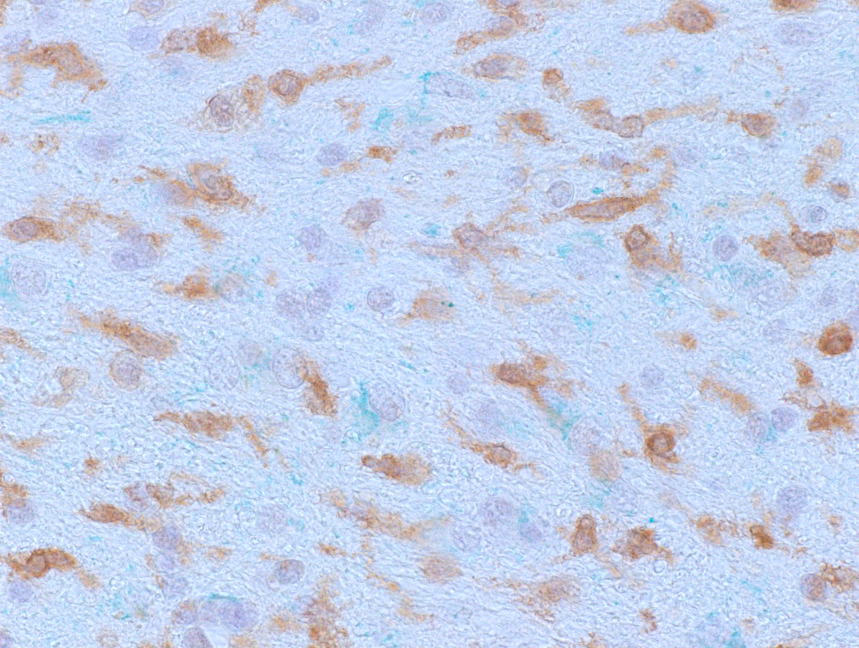


**#7 patient**


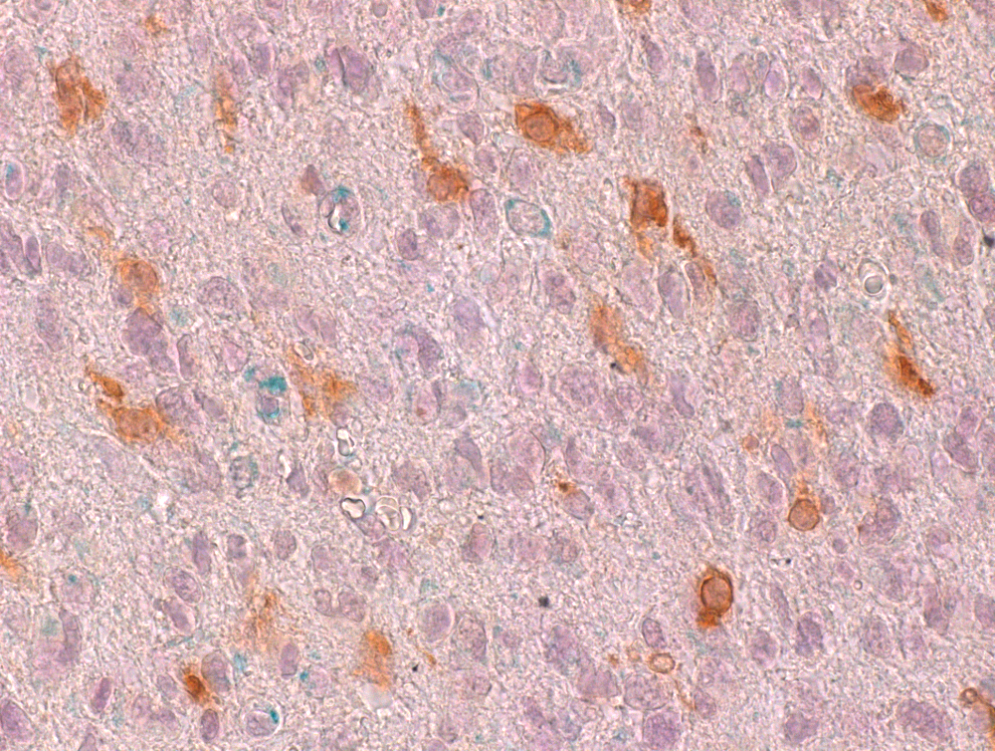

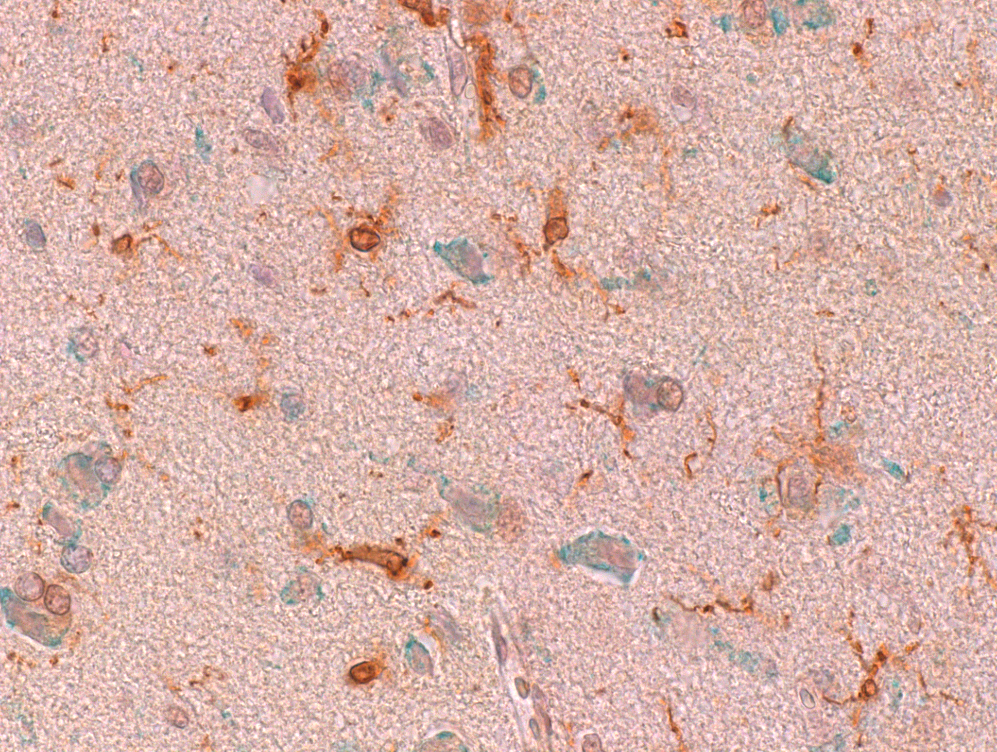


**#8 patient**


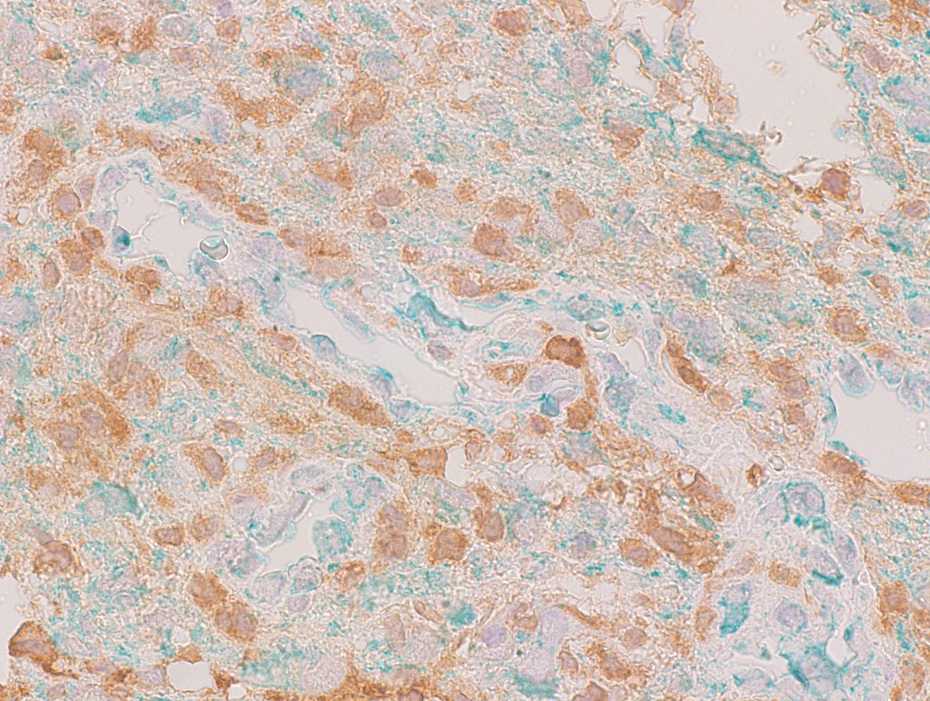

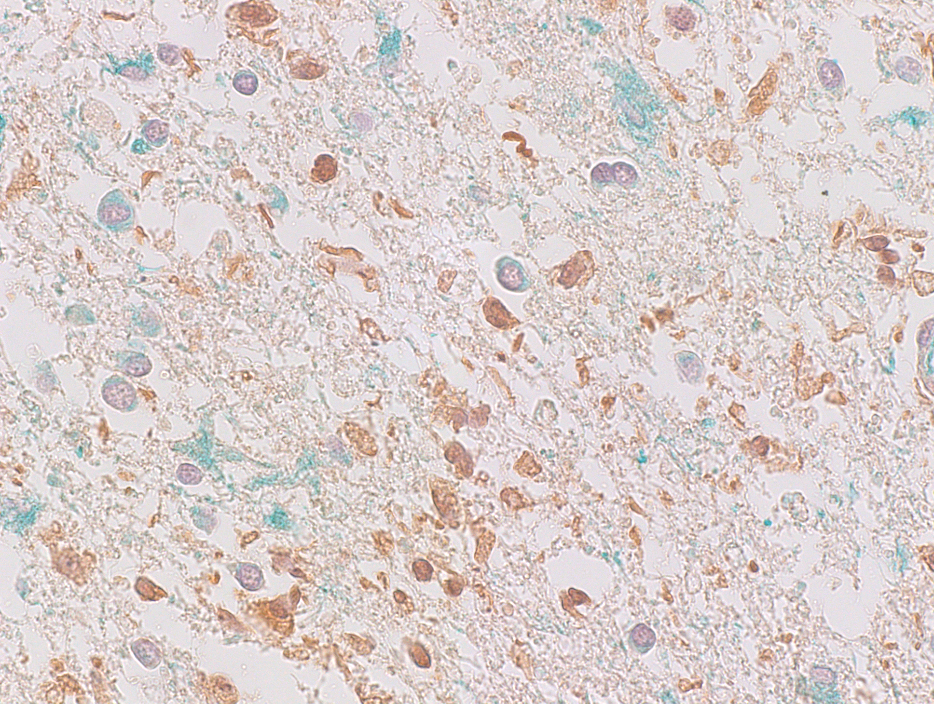


**#9 patient**


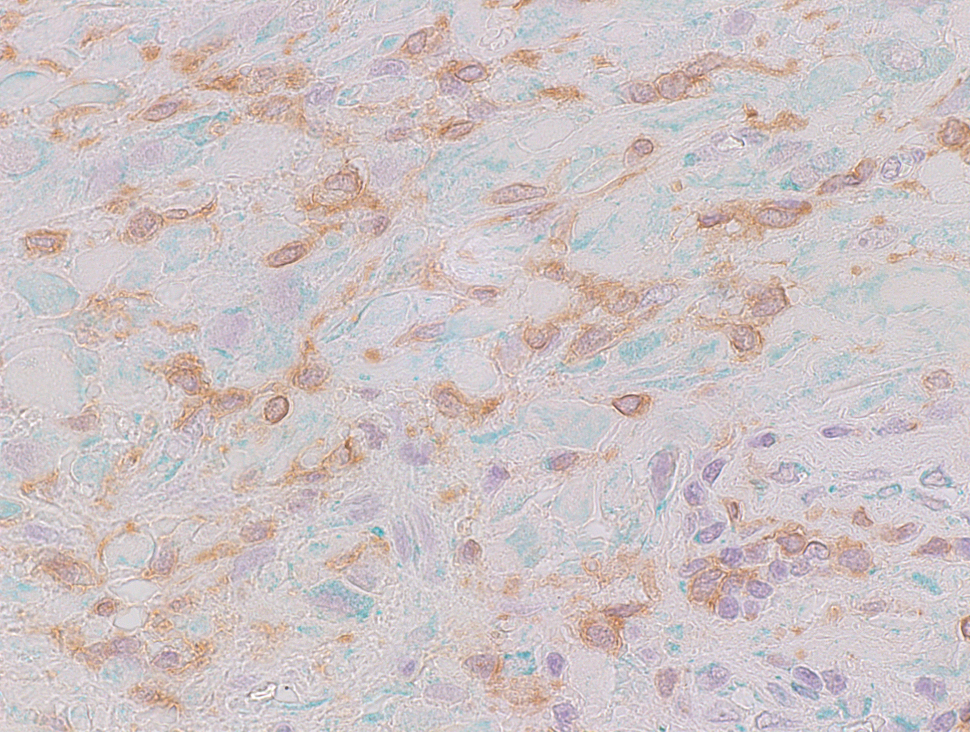

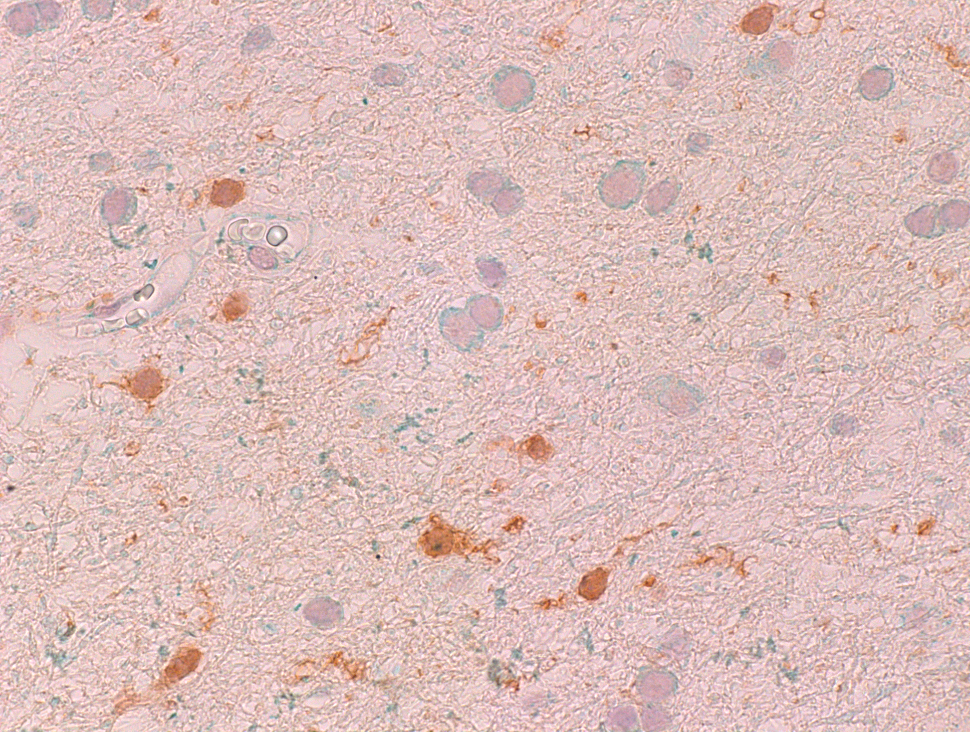


**#10 patient**


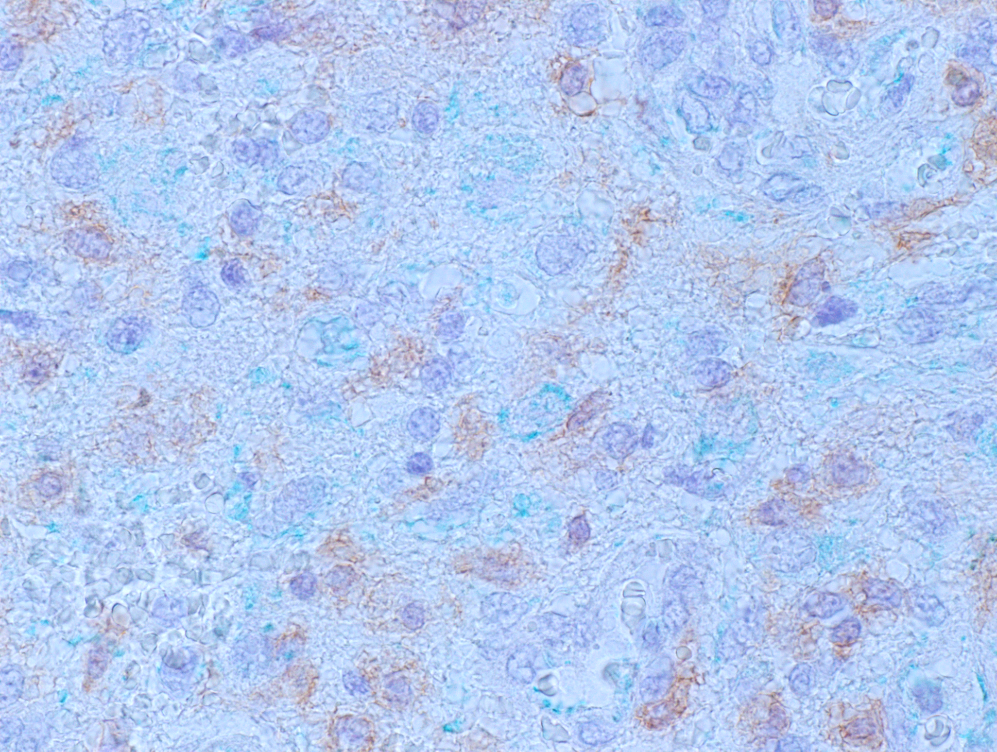

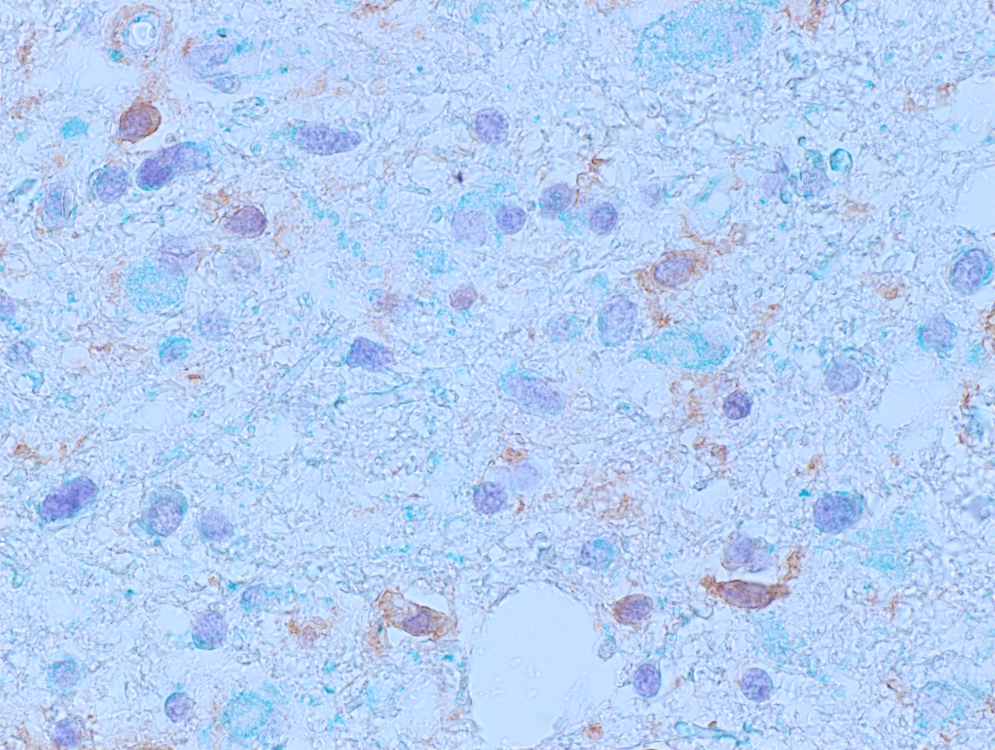


**#11 patient**


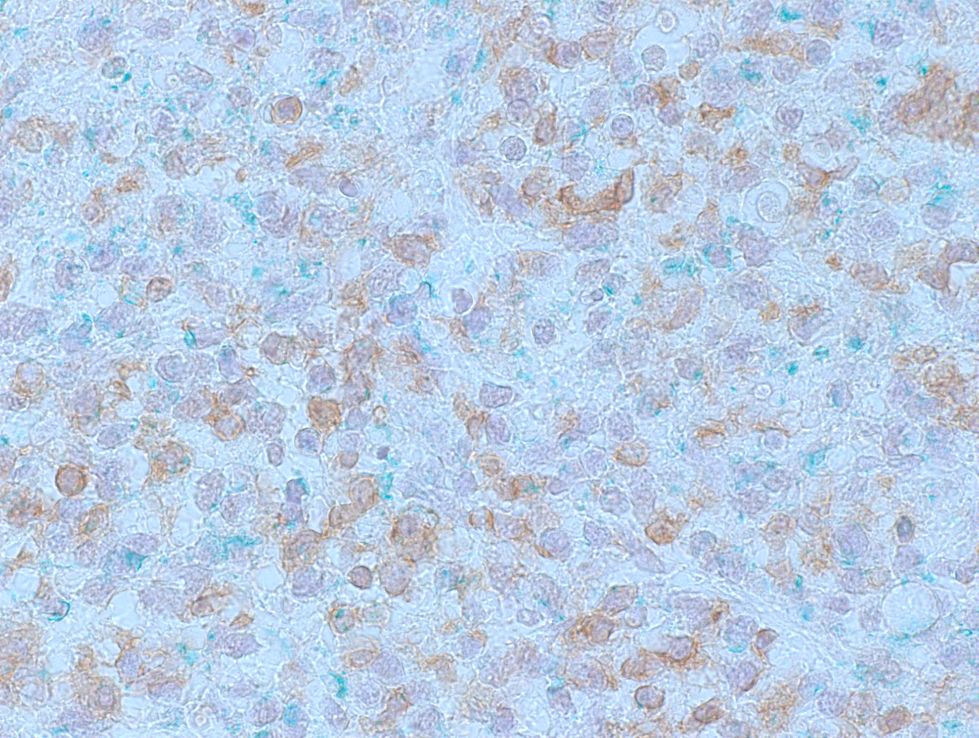

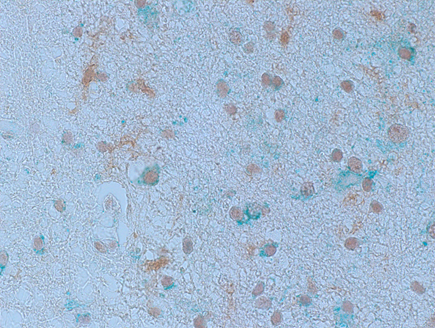


**#12 patient**


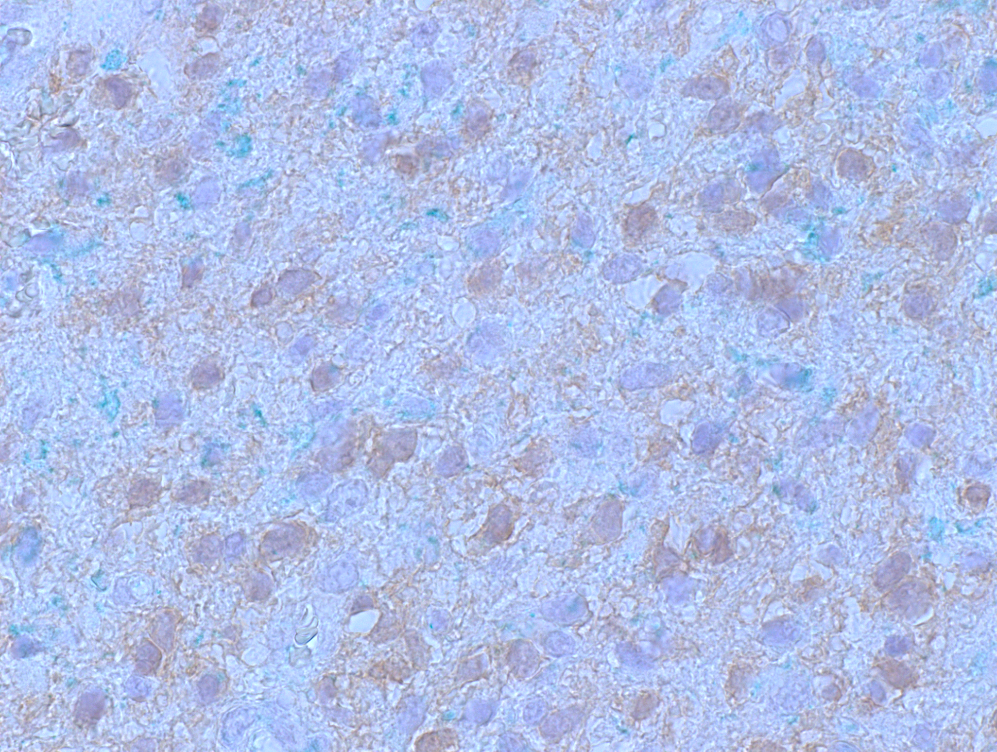

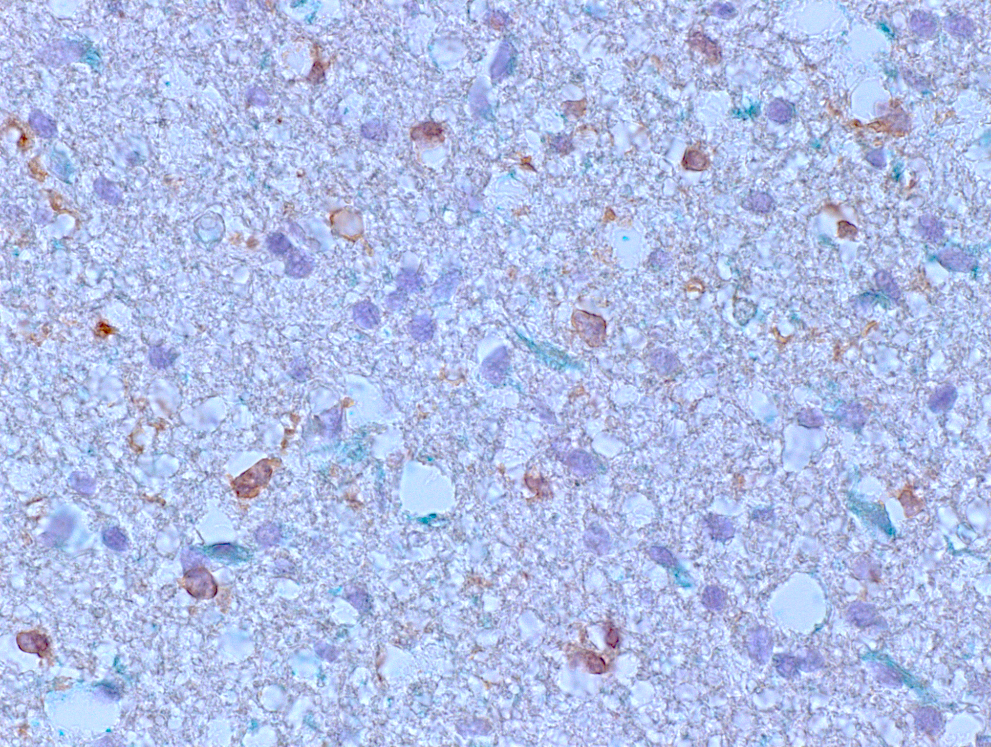


**#13 patient**


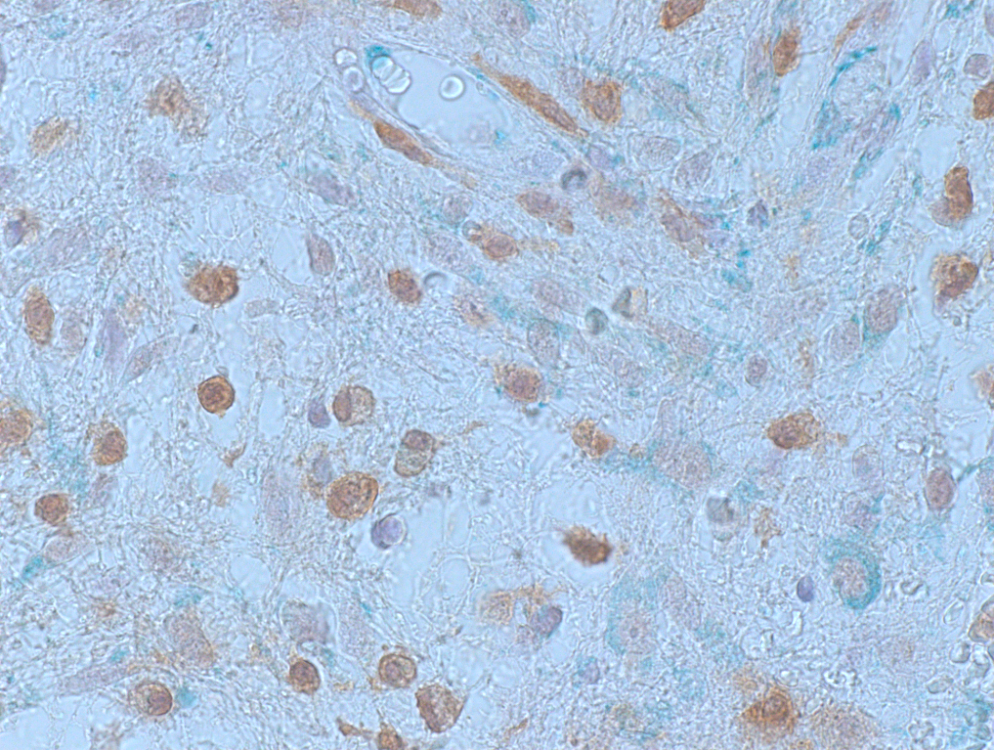

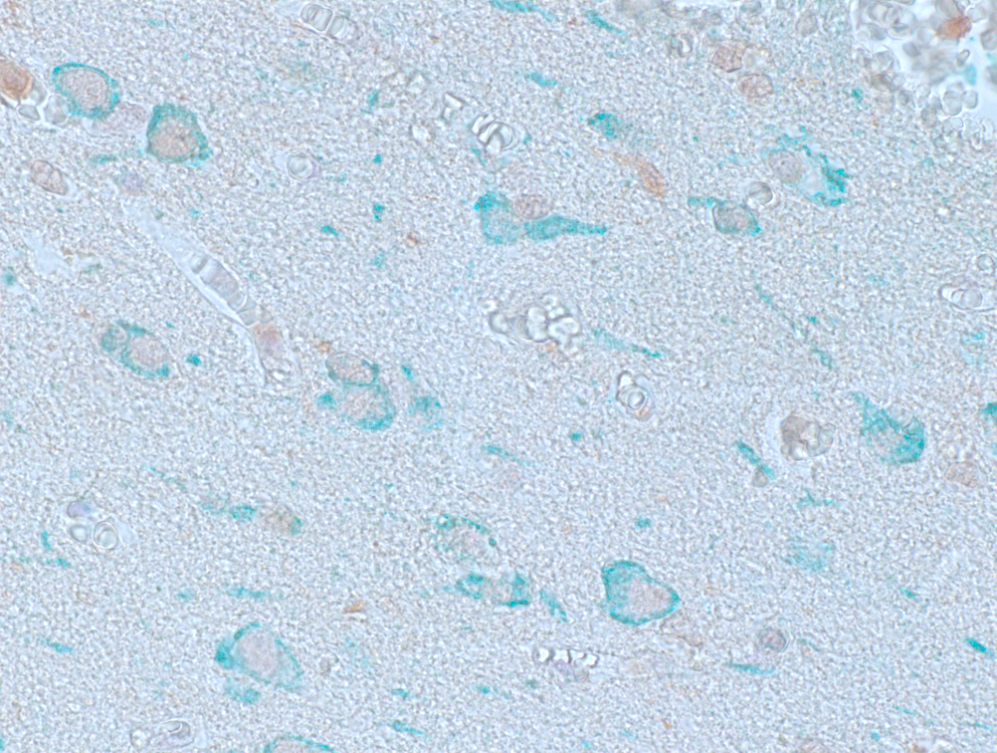


**#14 patient**


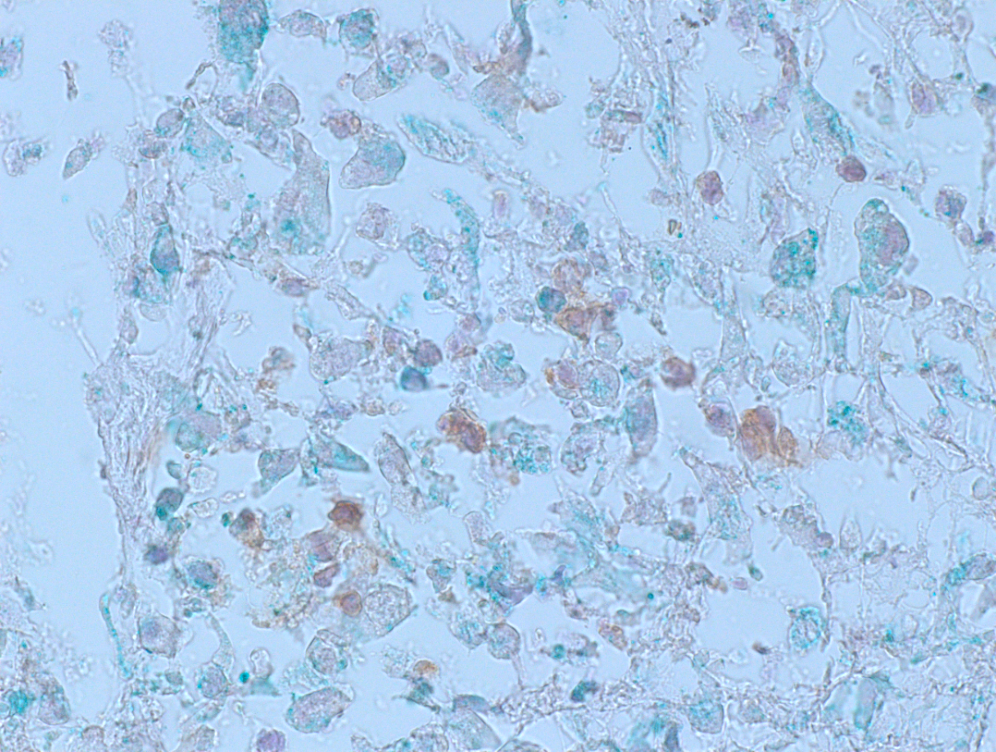

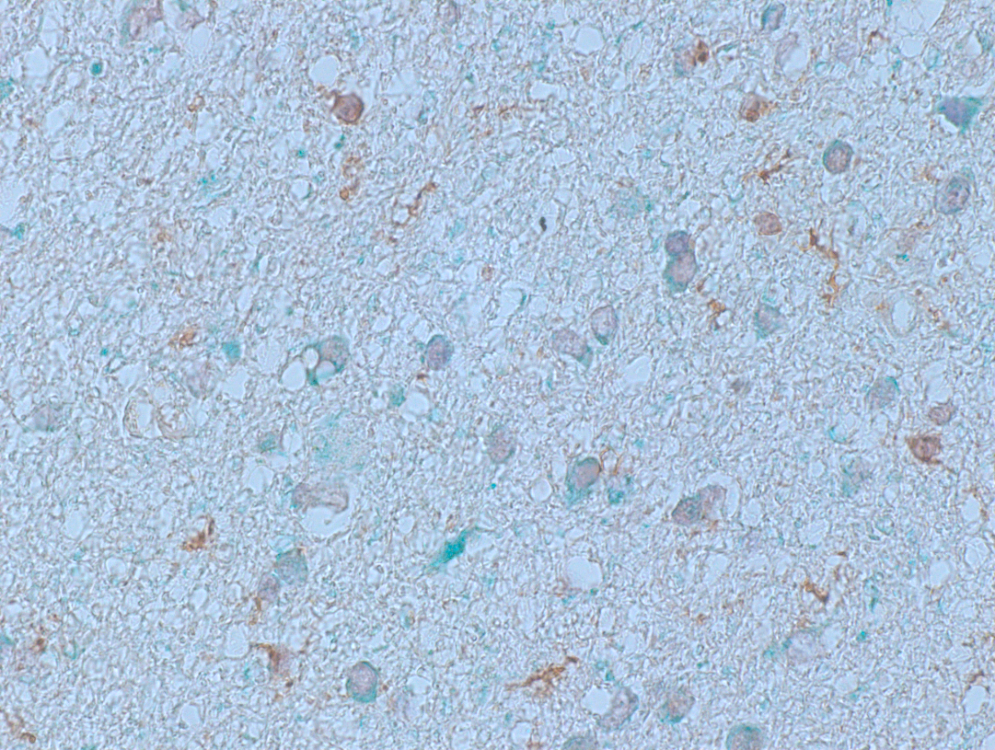


**#15 patient**


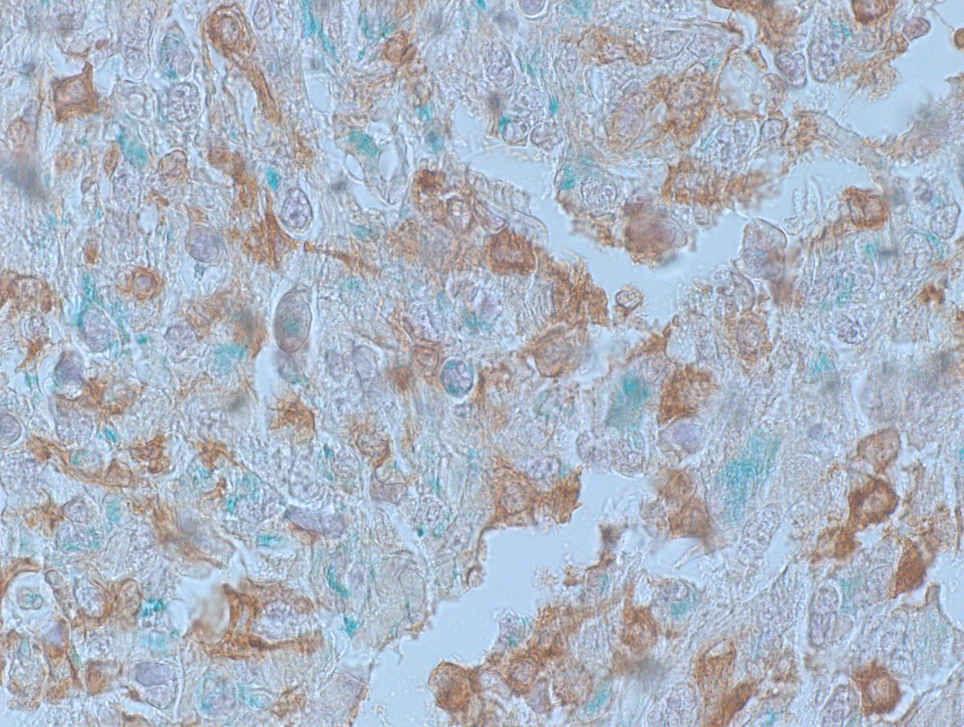

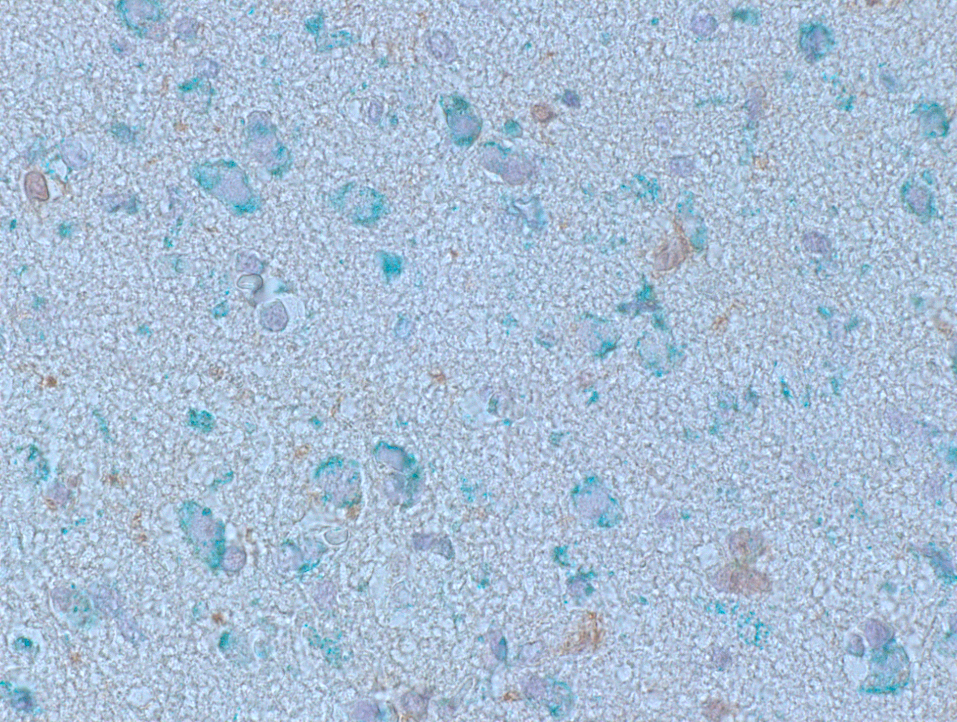


**#16 patient**


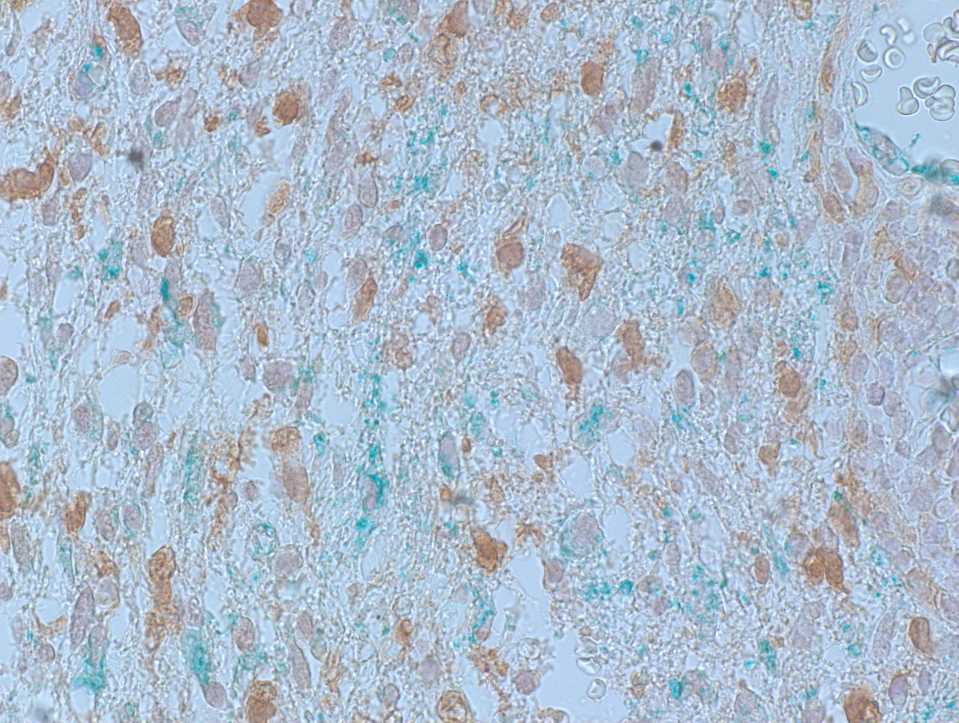

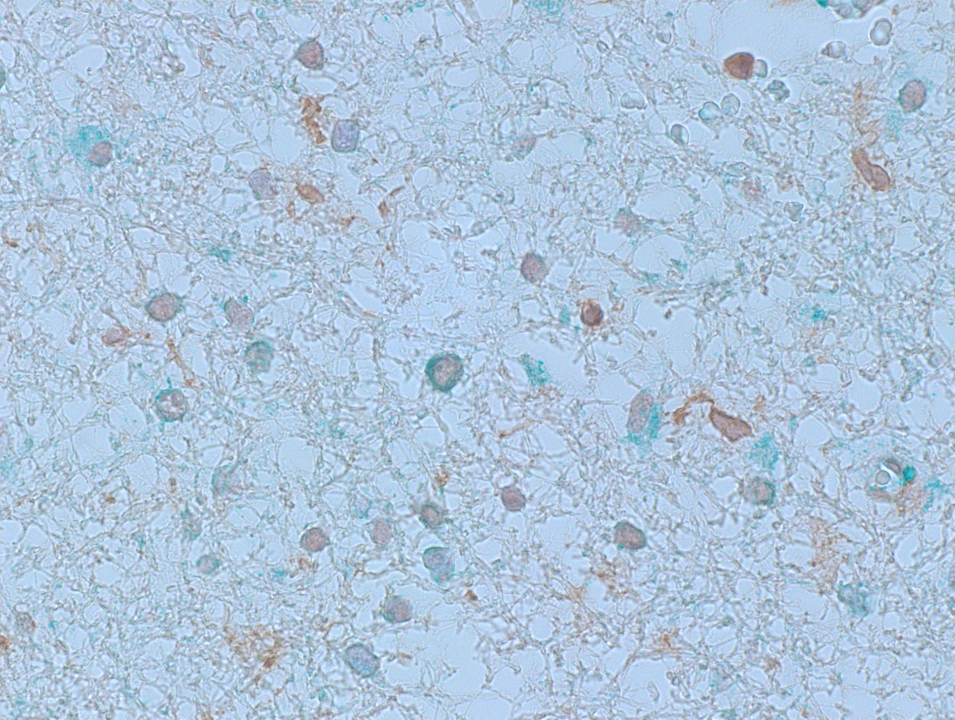


**#17 patient**


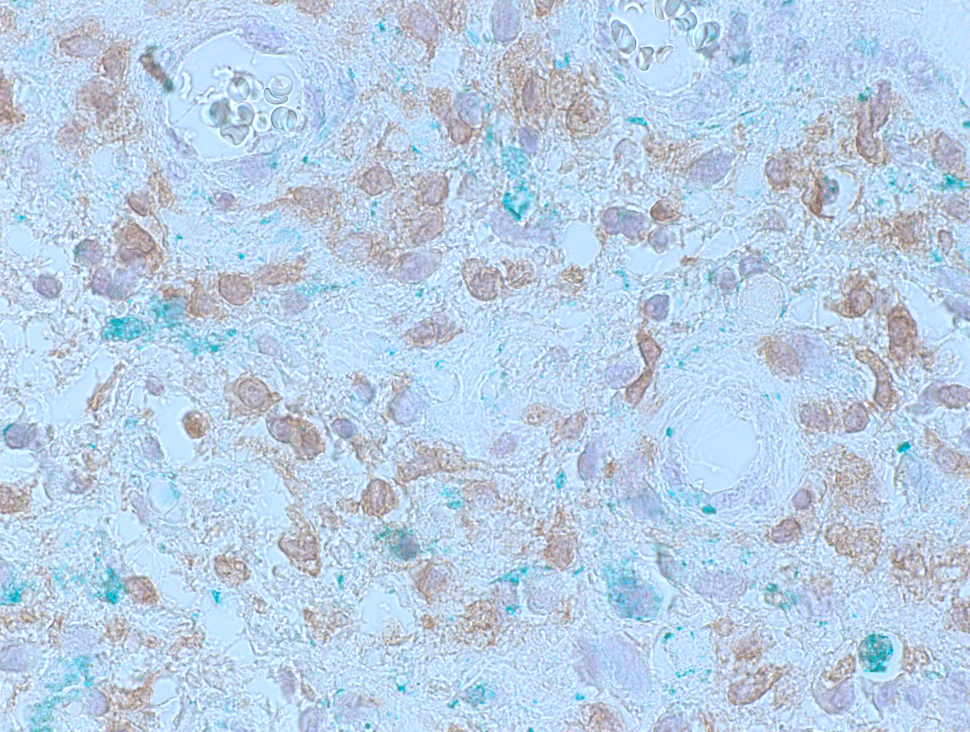

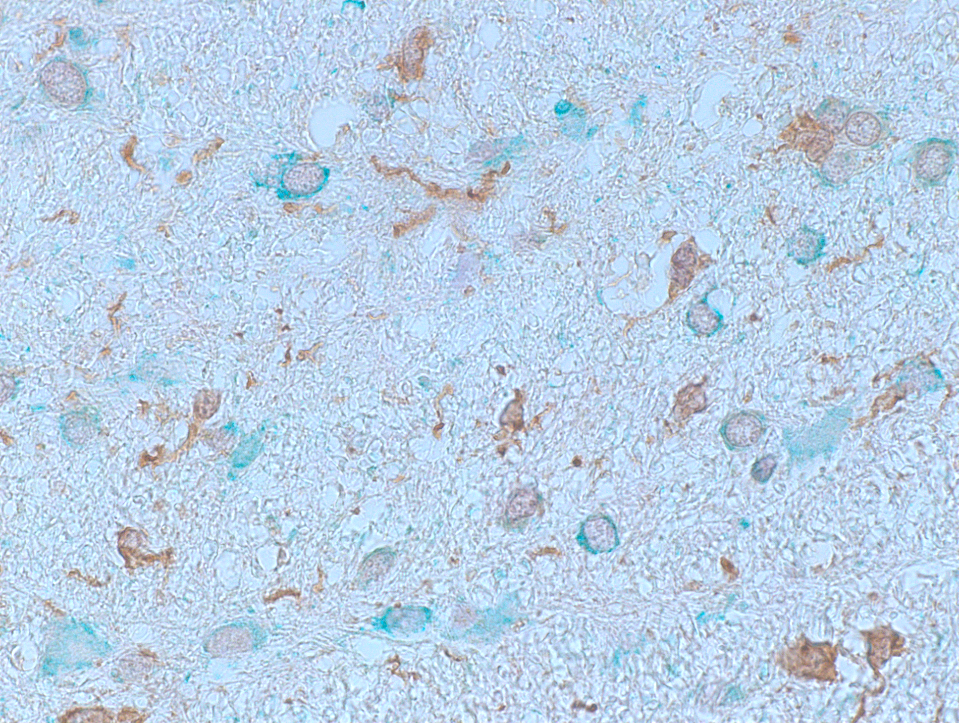


**#18 patient**


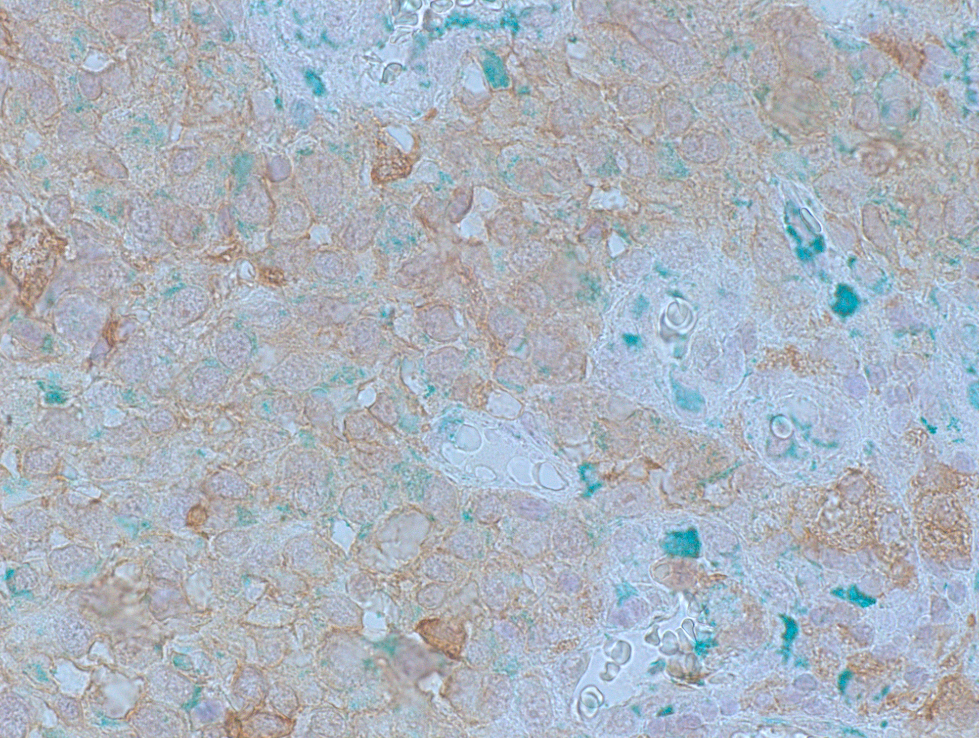

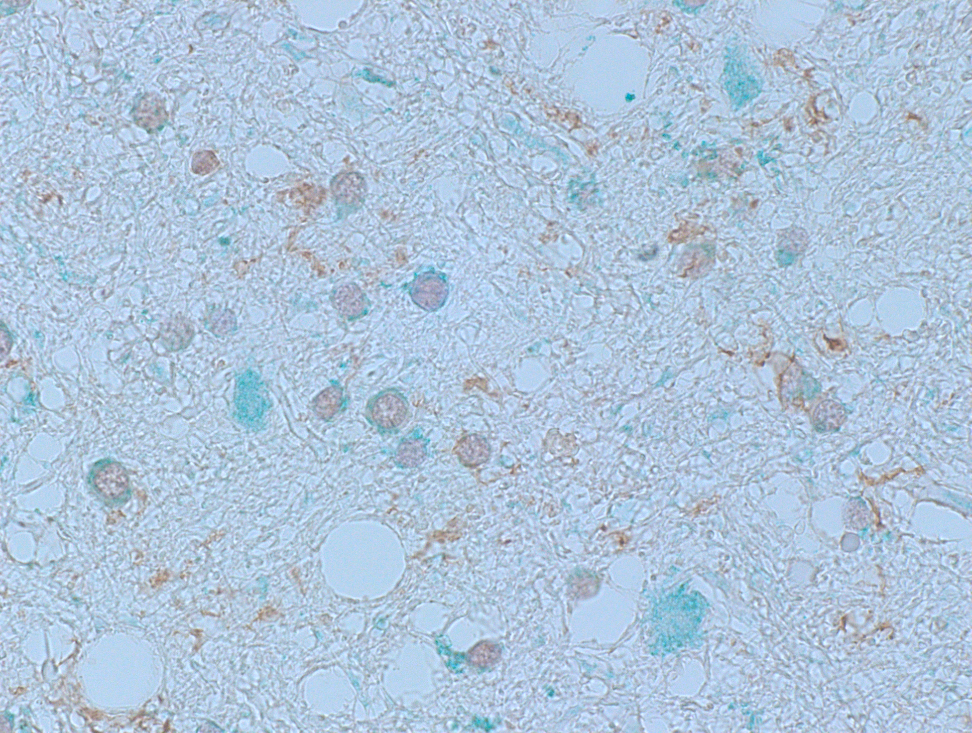


**Figure legend:**

Figure S1. PDIA3/IBA1 double staining in GBM specimens from 18 patients. Photographs in the left panels and right panels show representative tumor and parenchyma fields, respectively. The brown staining indicates IBA1 positive cells and the green staining indicates the PDIA3 positive cells. (magnification: 60x)
