# Supplementary material for: PDIA3 Expression in Glioblastoma Modulates Macrophage/Microglia Pro-Tumor Activation
Source: Int J Mol Sci. 2020 Nov 3;21(21):8214. doi: 10.3390/ijms21218214 (PMC7662700; doi:10.3390/ijms21218214)
Supplement: Supplementary file 1 [file ijms-21-08214-s001.zip › ijms-966417-revised-supplementary/Figure S2 rev.docx]

**Additional file 2**

**Figure S2**

**A**

**B**

**
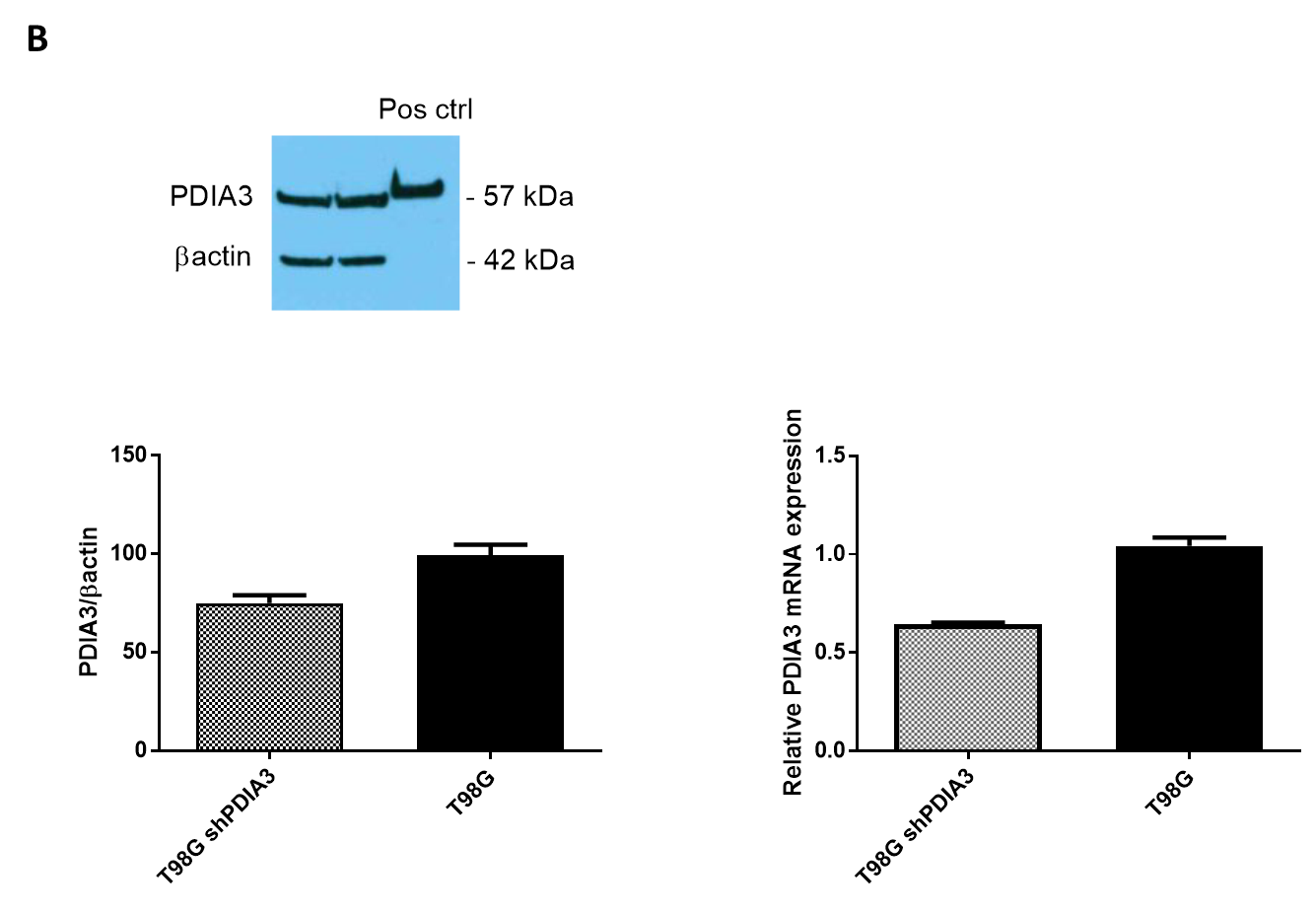
**

**C**

**
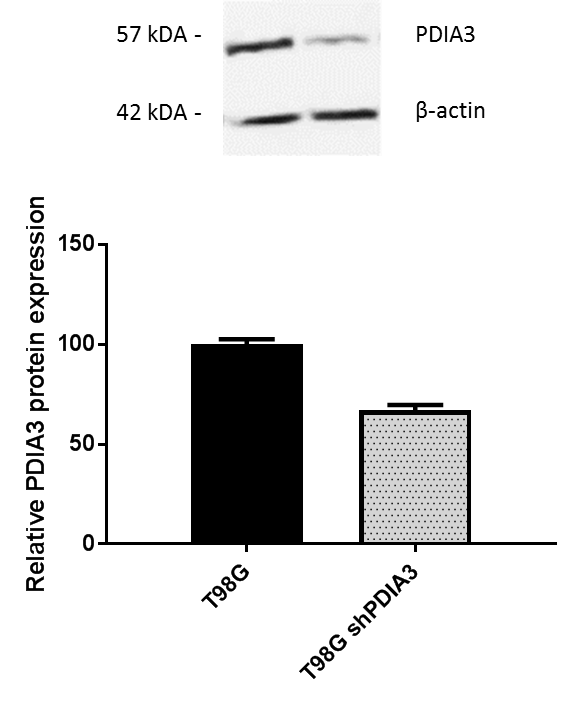
**

**Figure legend:**

**Figure S2.** PDIA3 gene expression and silencing in GB cells. A) PDIA3 mRNA expression in T98G and U87MG cells. B) PDIA3 mRNA expression in control and PDIA3-silenced T98G cells. C) PDIA3 protein levels in control and PDIA3-silenced T98G cells. In silenced cells, PDIA3 mRNA and protein levels were decreased about 35%.
