# Supplementary material for: PDIA3 Expression in Glioblastoma Modulates Macrophage/Microglia Pro-Tumor Activation
Source: Int J Mol Sci. 2020 Nov 3;21(21):8214. doi: 10.3390/ijms21218214 (PMC7662700; doi:10.3390/ijms21218214)
Supplement: Supplementary file 1 [file ijms-21-08214-s001.zip › ijms-966417-revised-supplementary/Figure S3.docx]

**Additional file 3**

**Figure S3**

**A**


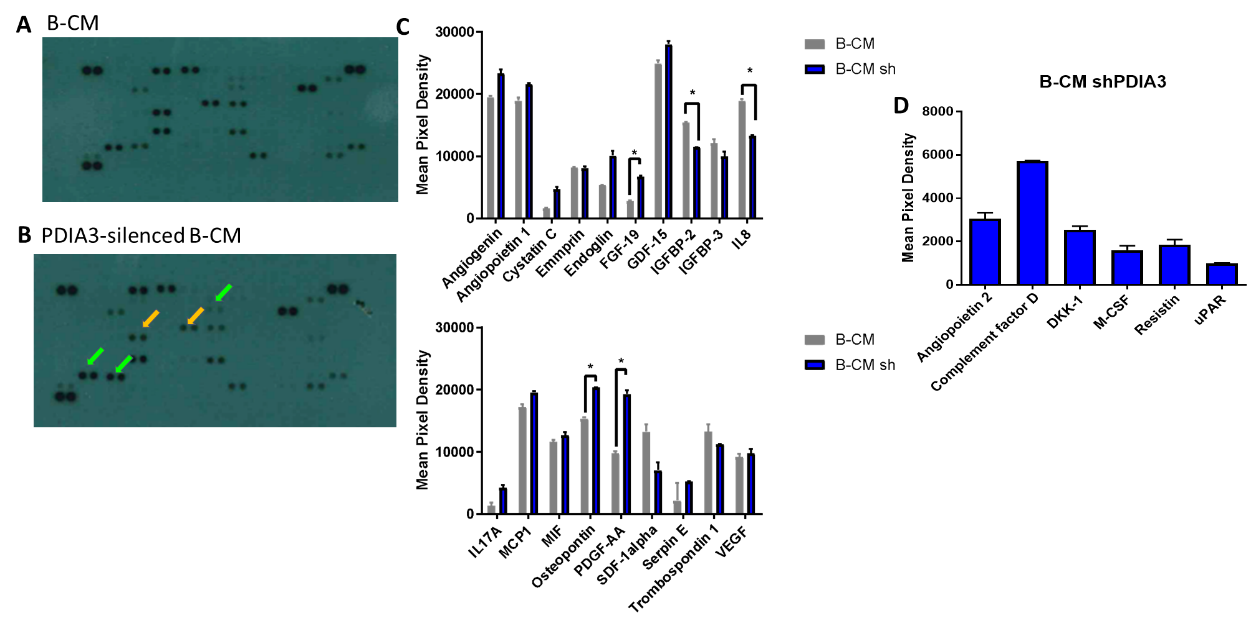


**B**


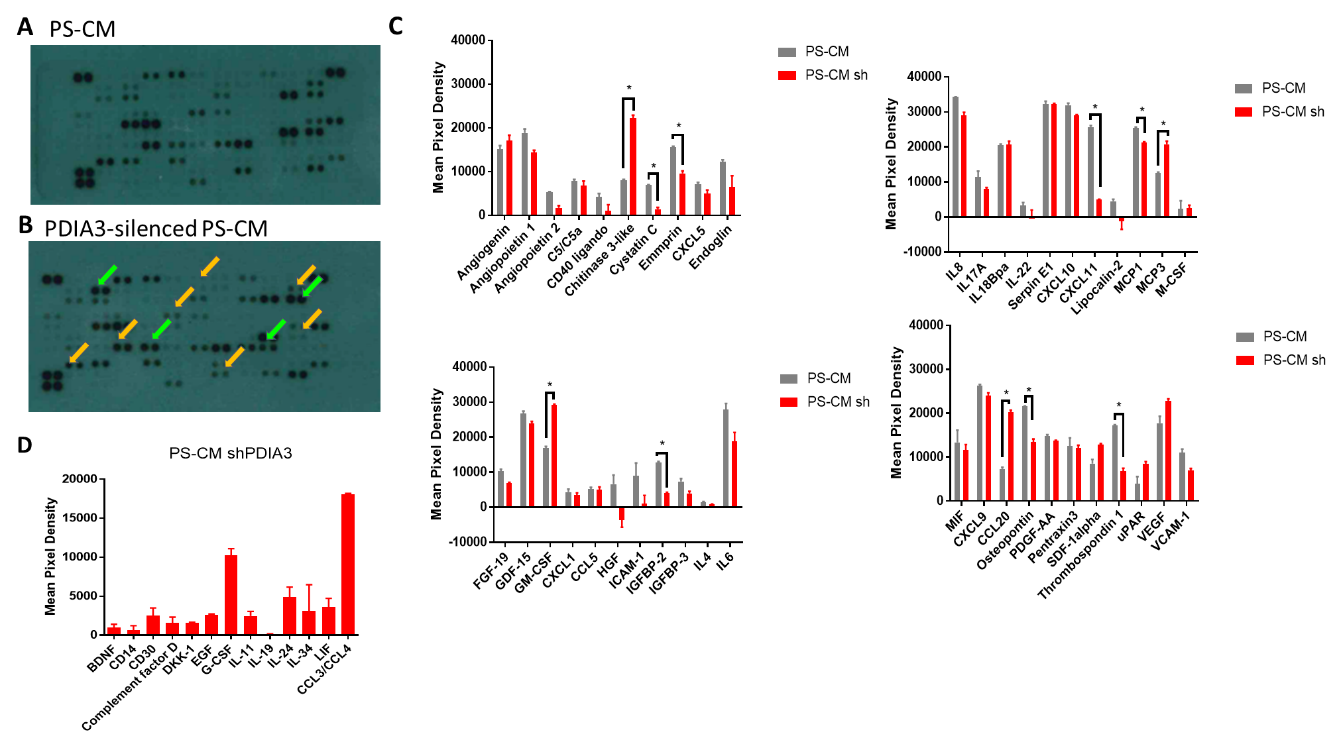


**Figure legend:**

**Figure S3.** Detail of all proteins that are modified in conditioned media. In particular, in panel A all the factors modified in the B-CM and in the B-CMsh, while in panel B all the factors modified in the PS-CM and PS-CMsh are indicated
