# Supplementary material for: PDIA3 Expression in Glioblastoma Modulates Macrophage/Microglia Pro-Tumor Activation
Source: Int J Mol Sci. 2020 Nov 3;21(21):8214. doi: 10.3390/ijms21218214 (PMC7662700; doi:10.3390/ijms21218214)
Supplement: Supplementary file 1 [file ijms-21-08214-s001.zip › ijms-966417-revised-supplementary/Figure S4.docx]

**Additional file 4**

**Figure S4**

**Figure legend:**

Figure S4. Reduction of nitrite levels elicited by punicalagin on CHME-5 cells. CHME-5 cells were treated for 48 hours with proinflammatory cytokines mix (TII) alone and in association with serial dilutions of punicalagin (PUN). Data are represented as mean ± SEM. **** p<0.0001.
